# Effects of Fumed Silica and Draw Ratio on Nanocomposite Polypropylene Fibers

**DOI:** 10.3390/polym9020041

**Published:** 2017-01-28

**Authors:** Luca Fambri, Izabela Dabrowska, Riccardo Ceccato, Alessandro Pegoretti

**Affiliations:** 1 Department of Industrial Engineering, University of Trento, via Sommarive 9, 38123 Trento, Italy; izabela_dabrowska84@wp.pl (I.D.); riccardo.ceccato@unitn.it (R.C.); alessandro.pegoretti@unitn.it (A.P.); 2 National Interuniversity Consortium for Science and Technology of Materials (INSTM), Via G. Giusti 9, 50121 Firenze, Italy

**Keywords:** fumed silica, fibers, drawing, polypropylene, XRD, nanocomposites, draw ratio, factorial experimental design, Pareto chart

## Abstract

Hydrophylic fumed silica AR974 was tested as a potential nanofiller for the production of composite isotactic polypropylene filaments/fibers (containing 0.25–2 vol % of nanoparticles) via melt compounding and subsequent hot drawing. The objectives of this study were as follows: (i) to investigate the effects of the composition and the processing conditions on the microstructure and the thermal and mechanical properties of the produced fibers; (ii) to separate the effects of silica addition from those produced by fiber drawing; and (iii) to interpret the changes in the matrix molecular mobility (produced by silica and/or drawing). Scanning electron microscopy (SEM) evidenced a good dispersion of nanoparticles at fractions up to 0.5 vol % of the nanofiller. X-ray diffraction (XRD) analyses revealed the increase in crystallinity after drawing of both neat polypropylene (PP) and produced nanocomposite fibers. Consequently, tensile modulus and stress at break of the fibers were enhanced. Drawn fibers containing 0.25–0.5 vol % of nanofiller showed also a remarkable increase in the creep resistance. Loss modulus of drawn fibers showed a pronounced α-relaxation peak at about 65 °C; the higher the draw ratio, the higher the peak intensity. Thermal and mechanical properties of composite fibers were improved due to the combined effects of nanofiller reinforcement and fiber orientation produced during hot drawing. Both fumed silica and draw ratio were significantly effective on tensile modulus and tenacity of nanocomposite fibers up to 0.5 vol % of AR974.

## 1. Introduction

”Fiber” is defined as “a unit of matter characterized by flexibility, fineness and high ratio of length to thickness” [1]. For successful application of fibers, market requirements encompass adequate mechanical, chemical and thermal stability. In particular, fibers have found applications in clothing and furnishing, but they are also widely used in various industrial sectors such as insulation, composites, geotextiles and filtration. The fibers traditionally used for textile applications such as clothing, household goods and some technical products are made of semi-crystalline linear polymers, due to their possibility to be molecularly oriented during processing. Most industrially produced synthetic fibers belong to the one of the following four chemical types: polyamide, polyester, polyvinyl and polyolefin [2]. On the market volume basis, polypropylene (PP) fibers occupy the fourth position and are expected to rise by 5.8% per year by 2021 [3]. PP fibers have found many applications owing to their balanced physical and chemical properties, such as low density, high crystallinity, high stiffness or hardness, good chemical resistance of polymer [4,5], and a relatively easy spinning which makes it possible to achieve very high extension of macromolecular chains and their maximum alignment [6,7]. The most common process for fiber productions consists of (i) the melt spinning in which fibers are extruded through a die and (ii) fiber drawing [8,9]. Adjustments of extrusion temperature, die size, winding speed and cooling temperature affect structure and properties of the as-spun fibers/filaments [10,11]. Afterwards, as-spun fibers are subjected to a large and almost irreversible elongation (producing parallel orientation of chains), which accounts for enhanced mechanical properties. If the neck propagates during drawing over the entire sample and the as-spun fiber is extended uniformly until break, the achieved draw ratio is called the natural draw ratio [12].

In the last decades, it has widely been proven that a small addition (<5% by wt) of inorganic nanoparticles like silica [13,14,15], carbon nanotubes [16], layered silicates [17,18,19], silver and titania [20,21] to polymeric matrices can profoundly improve physical properties of produced fibers, e.g., mechanical properties, gas barrier properties, fire retardance, antibacterial properties [14,17,20,21,22,23,24,25,26,27]. Silica has been largely used for improving PP properties, as documented by various papers dedicated to nanocomposite compounding by using either internal mixer [27,28,29,30,31] or melt extrusion [30,31,32], followed by injection molding or compression molding for characterization of bulk specimens. In order to improve the interaction between polypropylene and silica, either addition of compatibilizer, mainly maleic anhydride grafted polypropylene PP-*g*-MA, [28,31,33,34] or silica functionalization [25,35,36] have been reported in the literature, showing the effects of dependence on the type of polypropylene (homopolymer, copolymer, or blend), percentage of compatibilizer, filler content, and compounding process.

In our previous paper, we described the production of nanocomposite PP fibers containing fumed silica Aerosil^®^R805, i.e., a hydrophobic silica treated with octylsilane, and we have indicated optimum improvements in mechanical properties at the filler fraction 0.5% by vol. [15]. Following those previous results, the aim of the present work is to shed more light on the distinct effects of nanosilica content and of fiber drawing on the fiber properties. To this purpose, a different type of silica, i.e., hydrophobic silica Aerosil^®^R974 modified with dimethyldichlorosilane, was selected for the formulation of nanocomposite and for the production and characterization of polypropylene melt spun fibers. Besides, this nanofiller has higher specific surface area and higher bulk density then previously used [15] Aerosil^®^R805. Moreover, a commercial compatibilizer PP-*g*-MA was properly added to hydrophilic silica for evaluation of possible improvement of our PP/silica composites fibers. Our intention is the determination of key factors related to the processing conditions (nanofiller composition and draw ratio) in order to maximize the mechanical properties of fiber. Complementary information will be given by XRD analysis and dynamical mechanical measurements.

To the best of the authors’ knowledge, this paper represents the first study simultaneously describing the effect of the nanofiller content on the nanocomposite fiber and the effect of fiber drawing, taking into consideration statistical analysis.

## 2. Materials and Mehods

### 2.1. Materials

Fumed silica (FS), polypropylene, and maleic anhydride grafted polypropylene were selected as nanofiller, matrix and compatibilizer, respectively.

Aerosil^®^R974, hydrophobic silica (surface treated with dimethyldichlorosilane) was kindly supplied by Evonik (Essen, Germany). Fumed silica nanoparticles are characterized by a specific surface area of 170 m^2^/g, mean particle size of about 12 nm, and bulk density of 1.99 g/cm^3^ at 23 °C. Before processing, fumed nanosilica powders were dried for 24 h at 80 °C in a vacuum oven.

Moplen HP500, isotactic polypropylene with density 0.905 g/cm^3^ at 23 °C and melt flow rate of 1.8 g/10 min at 230 °C and 2.16 kg, was received from Lati SpA (Vedano Olona, Italy) in the form of pellets.

Fusabond^®^ P613, (PP-*g*-MA) maleic anhydride grafted polypropylene (maleic anhydride content = 0.25–0.50 wt %; melt flow rate 49 g/10 min at 190 °C and 1.0 kg; density 0.903 g/cm^3^ at 23 °C), was supplied by DuPont™ de Nemours (Geneva, Switzerland). This compatibilizer (C) was added to the composites with 0.5 vol % of fumed silica, whereby the ratio PP-*g*-MA/FS was 1:1 or 2:1 by vol. for preliminar trial of factorial design (see Table 1 and Section 2.4).

### 2.2. Compounding, Fiber Spinning and Drawing

Fibers were produced in a double step process (extrusion and hot-drawing) for compositions with 0.25 up to 2 vol % of fumed silica (Table 1). The compositions were selected in conformity with a preliminary study on compounding of AR974 with polypropylene followed by the characterization of nanocomposites plates produced by compression molding [37].

After compounding, the mixtures of PP and fumed silica were spun by a Thermo Haake PTW16 intermeshing co-rotating twin screw extruder (Thermo Haake, Karlsruhe, Germany), screw diameter 16 mm, L/D ratio 25, rod die diameter 1.65 mm) in order to produce fibers of about 500 µm diameter. The temperature profile from the hopper to the rod die was gradually increased in the range 130–230 °C. As-spun fibers were fast cooled in water at room temperature and wrapped around a rotating cylinder (40 mm diameter) rotating at 67 rpm.

Draw ratio (DR) is defined according to the following equation in dependence on initial (*S*_i_) and final (*S*_f_) fiber section:

(1) $$\mathrm{DR}=\frac{S_{i}}{S_{f}}\;={\left(\frac{D_{i}}{D_{f}}\right)}^{2}$$

where *D*_i_ and *D*_f_ are the initial and final diameters of the fiber. For instance, DR1 indicates as-spun or undrawn fibers, whereas the fibers drawn ten times are indicated with DR10.

Fiber hot-drawing was performed in air at 145 °C by using a hot-plate drawing apparatus 140 cm length (SSM-Giudici srl, Galbiate, LC, Italy) following the procedure described elsewhere [15]. Various drawn fibers were produced from a minimum draw ratio of DR4 up to a maximum value of about DR20, in dependence on the drawability of compounded nanocomposite.

The diameter of each single fiber specimen was measured as average of three measurements on digital pictures taken by an optical microscope and analyzed by the image processing software (ImageJ^®^), by using a calibration marker with accuracy ± 1 μm. The titer of fibers, *T*, or linear density, expressed in tex, is defined as the weight (in grams) of 1000 m of fiber following ASTM D681-07. It can be calculated from the fiber diameter according to Equation (2):

(2) $$T=\frac{d\;\pi }{1000}\;{\left(\frac{D}{2}\right)}^{2}$$

where *d* and *D* are the density and the diameter of the fiber, expressed in g/cm^3^ and micron, respectively. Nanocomposites were labeled with a code indicating the type of silica (AR974) and its volume percentage. For example, AR974-2 indicates a nanocomposite sample filled with 2 vol % of fumed silica Aerosil AR974. Neat polypropylene was designated as PP, and the samples of fibers containing maleic anhydride grafted polypropylene (PP-*g*-MA) were labeled with the code C (see Table 1).

### 2.3. Characterization Techniques

Melt flow index (MFI) measurements were performed by a Dynisco LMI 400 plastometer (Heilbron, Germany) according to ASTM D1238-10 at 230 °C and 2.16 kg. About 3 g of as-spun fibers were cut and inserted into the cylinder, where were preheated for 5 min before extrusion. Melt flow was expressed as average values of five measurements.

Quasi-static tensile mechanical properties of fibers were performed at room temperature by using an Instron ^®^4502 tensile testing machine (Norwood, MA, USA) equipped with a load cell of 100 N. Single filaments with diameters 0.5–0.1 mm were prepared using a thin paper test mounting tab with a gauge length of 30 mm fixing each specimen by using adhesive in order to minimize fiber misalignment, as recommended by ASTM C1557-03 (Standard Test Method for Tensile Strength and Young’s Modulus of Fibers), and were uniaxially tested at 50 mm/min up to break. Compliance of testing machine was neglected since its stiffness is much higher than the one of the tested fibers. The diameter of each single fiber specimen was considered to convert the tensile load into a tensile stress by using the Bluehill 3 Testing Software of the Instron machine. The elastic modulus was determined as a secant value between strain levels of 0.05% and 0.25%, according to ISO 527-1 standard. Strain was evaluated by normalizing the cross-head displacement over the gauge length of the samples. Fracture always occurred approximately in the center of the fiber. Tests have been performed on at least five specimens for each sample and the average values have been reported along with the standard deviation. Tenacity of fibers was calculated as the ratio between load at break and the titer.

Relative tenacity at constant draw ratio (RT_DR_) is calculated as the ratio of the tenacity of nanocomposites (*T*_AR974_) and the matrix tenacity (*T*_PP_) for each set of drawn fibers, i.e.,

(3) $$RT_{\mathrm{DR}}=\frac{T_{\mathrm{AR}974}}{T_{\mathrm{PP}}}$$

Relative tensile modulus at constant draw ratio (RTM_DR_) is calculated as the ratio of the modulus of nanocomposites (*E*_AR974_) and the matrix modulus (*E*_PP_) for each set of drawn fibers, i.e.:

(4) $$RTM_{\mathrm{DR}}=\frac{E_{\mathrm{AR}974}}{E_{\mathrm{PP}}}$$

Relative tenacity referred to polypropylene (*RT*_PP_) can be calculated as the ratio of the tenacity of nanocomposites (*T*_AR974_) and the matrix tenacity (*T*_PP_) at DR1, i.e.,

(5) $$RT_{\mathrm{PP}}=\frac{T_{\mathrm{AR}974}}{T_{\mathrm{PP}\left(\mathrm{DR}1\right)}}$$

Relative tensile modulus referred to polypropylene (*RTM*_PP_) is calculated as the ratio of the modulus of nanocomposites (*E*_AR974_) and the matrix modulus (*E*_PP_) at DR1, i.e.,

(6) $$RTM_{\mathrm{PP}}=\frac{E_{\mathrm{AR}974}}{E_{\mathrm{PP}\left(\mathrm{DR}1\right)}}$$

Creep response of the fibers was tested by DMA Q800 dynamometer (TA Instruments, New Castle, DE, USA) at 30 °C. Both unfilled PP and PP-silica nanocomposite fibers with a gauge length of 10 mm were tested for 3600 s under a constant stress of 3 MPa, corresponding to about 10% of the stress at yield of as-spun fiber [24]. The creep compliance *D*(t), computed as the ratio between the strain and the creep stress, was plotted against the logarithm of time. It shows the best fitting parameters of the Burgers model.

Thermogravimetric analysis (TGA) was performed in an air flow (25 mL/min) with fiber specimens of about 10 mg by using a TGA Q5000 IR (TA Instruments) equipment at a heating rate of 10 °C/min in the range 50–600 °C. The results represent the average of three tests. The rate of thermo-oxidation was evaluated at the maximum of the peak of derivative curve DTGA.

Scanning electron micrographs (SEM) were taken by a Philips XL30 environmental scanning electron microscope, (Philips, Eindhoven, The Netherlands), at an acceleration voltage between 20 and 25 kV. Micrographs visualize the surface produced by crio-fracturing the fiber specimens after 60 mins of immersion in liquid nitrogen and metallization with platinum/palladium by using a sputtercoater QUORUM Q150T ES (Quorum Technologies Ltd, Laughton, UK).

X-ray diffraction (XRD) spectra were collected by using a Rigaku III D-Max diffractometer, (Rigaku Corporation, Tokio, Japan), in a θ–2θ Bragg-Brentano geometry with a graphite monochromator in the diffracted beam (monochromatic radiation CuKα line with λ = 1.54056 Å). The following parameters were adopted: scan range: 3°–40° in 2θ; sampling interval 0.05°; counting time: 5 s, as previously set in the characterization of polyethylene–hydrotalcite nanocomposite fibers [38]. Fibers were tightly rolled up on an aluminum sample holder (~0.5 × 2 cm^2^) mounted orthogonal to the incident beam. As a rough approximation, the same areas of the samples were irradiated. Experimental spectra were handled in order to evaluate crystallographic features of the samples using a Jade 8^®^ software (MDI—Materials Data, Livermore, CA, USA). Crystallinity, *X*c, of the samples was calculated using the equation:

(7) $$Xc=\frac{A_{\mathrm{cr}}}{A_{\mathrm{cr}}+A_{\mathrm{am}}}100/f$$

where *A*_cr_ and *A*_am_ are the areas under the crystalline peaks and the amorphous halo, respectively, and f is the volume fraction of polymer matrix. Area values were calculated by a deconvolution step in the range 5°–30° of the diffraction spectra. Moreover, crystallite size dimensions for the more intense reflexes, *L*_hkl_, were evaluated by means of the Scherrer equation [39]:

(8) $$L=L_{\mathrm{hkl}}=\frac{0.9\;\lambda }{\beta \cos\theta }$$

where λ is the monochromatic X-ray wavelength, θ is the incident angle of the radiation to the surface of the sample and β is the integral breadth at half maximum of the referred peak [40].

Dynamic mechanical thermal analysis (DMTA) was carried out by DMA Q800 (TA Instruments, New Castle, DE, USA) in tensile mode in the temperature interval from −125 to 125 °C with a heating rate of 3 °C/min by using a fiber clamp (gauge length of 10 mm; pre-stress of 0.01 N; sinusoidal strain with a frequency of 1 Hz and amplitude of 64 microns). Storage modulus and loss modulus of as-spun and selected drawn fibers were measured and compared as function of temperature.

### 2.4. Statistical Analysis

In order to evaluate the effects of material composition and processing on mechanical properties of fibers, two series of fiber samples were prepared and tested. The obtained experimental results were compared in terms of analysis of variance, effects plots and factorial analysis, following the section Design of Experiments (DoE) utility of the Software Minitab 17, release 3.1.0, (Minitab Inc., State College, PA, USA; www.minitab.com/en-us/products/minitab/). In this way, a quantitative index, the *F*-value was generated for evaluating the significance of the effects of the investigated factors, within a chosen level of risk, α = 0.05, as commonly used in the literature. From the *F*-value and the probability *P*, the significance of each effect is then determined and compared.

Factorial design approach in Series I has been applied with three factors (compatibilizer amount, draw ratio, and fumed silica content) in order to determine seven coefficients (three linear; three at two-way interactions; one at three-way interactions). In Series II, only two factors were considered, i.e., draw ratio and fumed silica content, and three coefficients (two linear; one at two-way interactions) have been evaluated. Statistical results are presented by means of the Pareto chart and of the Normal plot of the standardized effects. More details of statistical analysis are reported in Supplementary Materials.

## 3. Results and Discussion

As-spun fibers of neat and nanofilled polypropylene with or without compatibilizer are summarized in Table 1. Melt flow analysis was performed in order to evaluate the effect of twin-screw extrusion on polymer matrix and on compounded nanocomposites. Subsequently, fibers were drawn at different draw ratios and characterized as described and discussed in the following paragraphs.

### 3.1. Melt Flow Index of the Prepared Polypropylene (PP) Composites

Table 1 evidences the melt flow index (MFI) after melt extrusion of various compositions. PP exhibits an almost constant value with respect to the technical data sheet declared by the producer. On the other hand, the addition of fumed silica determined a slight increase of melt flow that appeared directly proportional to the volume fraction of filler. A first interpretation is the attribution of experimental results to some thermal degradation of the PP matrix during processing, in analogy with the previous description by Dorigato after 15 min of melt compounding [41]. However, in that case, the increase of melt flow was attributed to the radical thermo-oxidation due to presence of oxygen in the chamber of internal mixer, and it was more markedly observed in neat polypropylene than in nanocomposite with fumed silica at 2% by vol. On the other hand, in the case of melt compounding in twin-screw extrusion described in this study, the amount of oxygen is certainly negligible, and the linear increase of melt flow of compounded nanocomposites could be attributed to the effect of organic layer of functionalized silica that could behave as internal lubricant, and consequently reduce the viscosity.

Moreover, as expected, for the compositions with 0.5 and 1.0 vol % of PP-*g*-MA, the significant increase of MFI can be ascribed to the higher MFI of the compatibilizer. A similar result was observed by Lee and Youn in polypropylene/layered-silicate nanocomposites, and it was considered a negative effect because it reduced the macromolecular orientation during melt spinning of fiber [42].

### 3.2. Tensile Mechanical Properties

Evaluation of mechanical properties is the crucial point in composites and fiber production. Both as-spun and drawn fibers were extensively tested and compared as function of draw ratio (DR).

Two series of experimental trials were performed taking into consideration the role of compatibilizer, the nanofiller content, and the draw ratio.

The first experimental trials (Series I) were conducted using factorial design with or without nanofiller (0.5 vol %), at different contents of compatibilizer (C) and at various drawing ratios.

In Series I of the Design of Experiment, to test the effect of the addition of PP-*g*-MA compatibilizer (C) on mechanical properties of composite fibers, the optimum composition with 0.5 vol % of fumed silica AR974 was selected, as starting composition following previous findings related to fumed silica AR805 [15]. A stronger stiffening effect can be expected [43] due to (i) an improved nanofiller/matrix interaction; and (ii) a more uniform nanofiller distribution in the polymer matrix.

**Figure 1:**
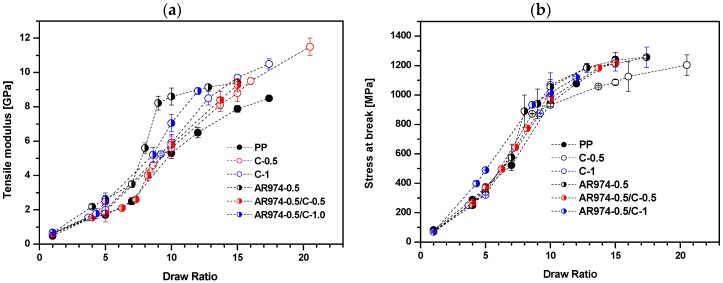
Effect of PP-*g*-MA on tensile modulus (**a**); and tensile stress at break (**b**) of PP fibers at different contents of compatibilizer (C), 0%, 0.5% and 1%, with and without 0.5% of fumed silica AR974 (percentage by volume).

Figure 1 shows tensile modulus and stress at break values of as-spun fiber and drawn fiber. The higher the draw ratio, the higher the fiber orientation, and the higher the modulus and the stress at break values, (and the lower the strain at break). Tensile modulus of fibers with either fumed silica (AR974) or compatibilizer (C) or both AR974 and C, is higher than that of neat PP fibers (Figure 1a). These results suggest a combined stiffening effect of nanofiller and compatibilizer. However, the compatibilizer appears to act as a simple promoter of polymer chain alignment, being its molecular weight much lower (higher MFI) than that of PP. On the other hand, the effect of AR974 and C on stress at break does not manifest a clear tendency (Figure 1b); but a negative effect of compatibilizer can be still inferred. The obtained results show that addition of the compatibilizer (in the amounts of 0.5% and 1% by vol) did not enhance the tensile modulus and stress at break of the nanocomposite fibers filled with 0.5 vol % of the fumed silica. It is worthwhile to note that at low draw ratios the strain at break of composites containing the compatibilizer is slightly higher than AR974-0.5 without compatibilizer. However, for DR10-15, the difference seems to become less significant and at the highest, DR is almost negligible.

All tensile modulus and stress at break data were interpreted following the Design of Experiment analysis, as documented detail in Table S1, Supplementary Materials. A factorial design approach and factorial regression was evaluated considering three linear terms, three two-way interaction terms and one three-way interaction term. The analysis of variance of tensile modulus as well as coded coefficients, regression equation and fits/diagnostics are presented in Table 2, and Tables S2–S4, Suppplementary Materials. The analogous results of factorial regression of stress at break are shown in Tables S5–S7, Supplementary Materials. As reported in the tables, the two terms Draw Ratio and AR974 were effective with probability value more than 99%, being *p*-value ≤ 0.004.

On the other hand, the probability value relative to the effect of compatibilizer (C) was 0.44–0.45 for both the variance analyses, which is more than 0.05, so this term had no effect on the trend of tensile modulus and stress at break. Moreover, the interaction among draw ratio and compatibilizer was not effective according to their probability values, *p* = 0.45, for both tensile modulus and stress at break. The interaction among draw ratio and fumed silica (AR974) was the only significant interaction on tensile modulus with *p*-value = 0.010; lower effectiveness on stress at break was determined for the interaction among compatibilizer and draw ratio (*p*-value = 0.02). The interaction between the three terms is absolutely not effective for both tensile modulus (*p*-value = 0.33) and stress at break (*p*-value = 0.99).

**Figure 2:**
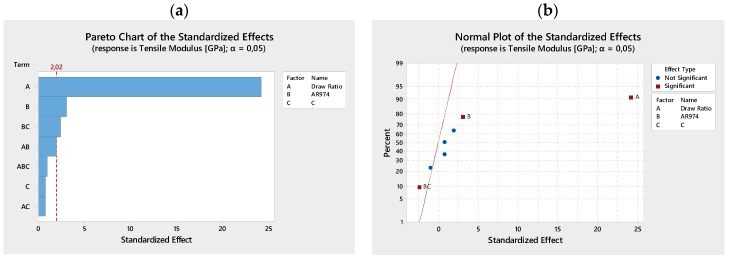
Series I. Pareto chart (**a**) and normal plot (**b**) of the standardized effects on tensile modulus. The significance of draw ratio and fumed silica, as main factors is shown.

**Figure 3:**
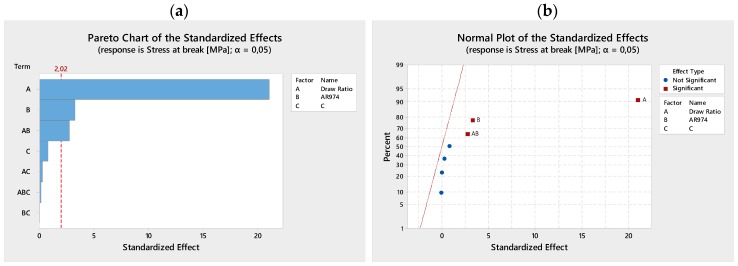
Series I. Pareto chart (**a**) and normal plot (**b**) of the standardized effects on stress at break. The significance of draw ratio and fumed silica, as main factors is shown.

Pareto charts (Figure 2a and Figure 3a) illustrate the effectiveness or not-effectiveness of analyzed terms (linear and interactive terms), in particular the draw ratio that is the most effective, and the nanofiller AR974. The normal plots of the standardized effects on tensile modulus (Figure 2b) and stress at break (Figure 3b) summarize the results of the factorial design analysis, where the two terms, draw ratio and fumed silica, are presented as significant, according to the chosen level of risk α = 0.05.

Selected contour plots of tensile modulus and stress at break are shown in Figures S1–S4, Supplementary Material. From statistical analysis of experimental trials of Series I, it is possible to conclude that the role of compatibilizer is not significant, and therefore the next experimental trials focused on two factors only: fumed silica content and draw ratio.

**Figure 4:**
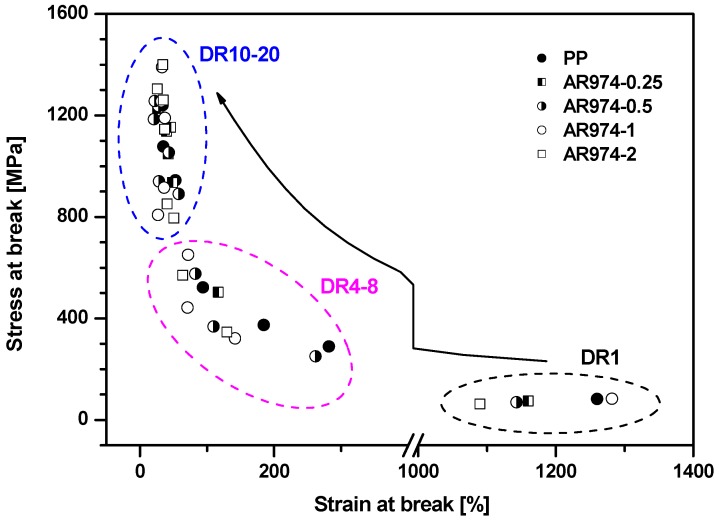
Representative comparison of stress at break as function of strain at break of selected neat and nanofiller PP fibers.

Figure 4 shows the relationship between stress at break and the corresponding deformation at break for selected drawn fibers at different draw ratios of polypropylene and nanocomposite fiber with AR974 at composition in the range 0.25 and 2 vol %. In particular, Figure 4 documents that the increase in stress at break is accompanied by decrease in the tensile strain at break as a consequence of drawing. The latter quantity decreases with the draw ratio from about 1250% for as-spun PP to 34%–32% for AR974-1 with DR15 (see also Table 3). Strain at break of as-spun fibers decreases with the fraction of incorporated fumed silica (Table 3), while after drawing process the difference between PP and nanofilled fibers diminishes so that the values achieved at the highest draw ratio DR15-DR20 are very similar.

**Figure 5:**
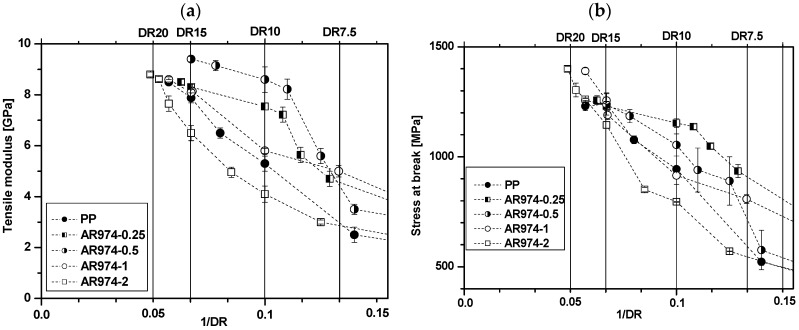
Tensile modulus (**a**) and stress at break (**b**) of the neat and nanocomposite PP fibers of Series II with different amount of fumed silica as function of draw ratio (DR).

Table 3 compares titer, tenacity and other mechanical properties of selected fibers with or without compatibilizer, whereas the results of tensile modulus and stress at break of all drawn fibers without compatibilizer are as shown in Figure 5a,b, respectively. Tenacity of fibers is calculated as the ratio between load at break and the titer.

Two parameters appeared of great relevance in nanocomposite fiber properties, i.e., the filler content and the drawing ratio, that will be considered and discussed in detail. Table 3 shows that the titer of the as-spun fibers varies from 174 tex of the neat matrix to about 179–186 tex of the nanocomposites, evidencing a direct dependence on the filler content, due to the higher density of fumed silica. The titer decreases with rising DR. PP and nanocomposite fibers of about 35–37 tex, 17 and 11 tex were produced via drawing to DR5, 10 and 15, respectively. Tenacity of as-spun fiber was found to decrease with the filler content, whereas in drawn fibers some positive effects on tenacity were evidenced for composition up to 1 vol % of fumed silica. For instance, nanocomposite fibers AR974-0.25 and AR974-0.50 with DR10 show higher tenacity (116–127 cN/tex) than corresponding PP fiber (104 cN/tex), while at DR15, both PP and nanocomposite fibers up to 1 vol % of nanofiller have tenacity of about 136–137 cN/tex.

Tensile modulus of nanofilled fibers increases with the percentage of fumed silica only up to 0.5 vol % for both as span (DR1) and drawn fibers, as shown in Table 3.

In the case of DR1, modulus of nanocomposite fibers is always higher than that of PP as span fibers, in conformity with the expected behavior of bulk composites. On the other hand, a peculiar tendency can be observed during fiber drawing, because the nanofiller seems to have a defect at 1%–2% vol., especially for DR 10 and DR15. At the higher draw ratio, the stiffening effect of polymer chain orientation appeared to prevail on fumed silica addition at high fumed silica content. The highest values 8.3 ± 0.5 and 9.4 ± 0.3 GPa at DR15 were achieved for AR974-0.25 and AR974-0.5 samples, respectively (7.9 ± 0.4 GPa was found for PP fiber). The stiffening effect, especially at low nanofiller amount, could be attributed to (i) even distribution of nanofiller particles in the matrix; and (ii) reduction of the mobility of macromolecules adhering to filler surface [25]. At elevated concentrations, nanofiller particles may form agglomerates (documented by the SEM and XRD analyses in the following paragraphs) that impair potential effects on increasing tenacity and stress at break, especially at higher DR.

It should be noted that existing literature evidences various dependencies of the stress at break of nanofilled PP fibers on filler content [14,15,16,17,23,24,25,26,43,44,45,46], i.e., either increasing, or insensitive, or even decreasing. Table 3 shows that stress at break of our samples is raised by fumed silica in the interval 0.25–0.5 vol %, while slightly lower values were found for the fibers with 2 vol % of filler.

Both draw ratio and filler content can affect the properties of the fiber. In general, the higher the draw ratio, the higher the modulus; the higher the stress at break, the lower the strain at break of the fiber. Moreover, the higher the filler content, the higher the modulus of composite materials. However, in the case of nanocomposite fibers, the modulus of drawn fiber is not always increased with the filler content (Table 4), because the combined effects of material composition (filler content) and processing conditions (compounding and especially drawing) are not directly cooperative.

An interesting approach for the evaluation of the draw ratio effect is shown in Figure 5, where tensile modulus and stress at break of high drawn fibers are reported as a function of the inverse of draw ratio. In this way, it is easy to visualize the tendency of a given property considering not only the drawing, but also the nanocomposite filler content.

In particular, the composition at low filler content (0.25% and 0.5% by vol.) seemed to show low modification at high draw ratio, whereas nanocomposite fibers with 1%–2% by vol. of fumed silica showed further possibility of improving both tensile modulus and stress at break, depending on the fumed silica dispersion, as evidenced in SEM micrographs of drawn fibers.

A comparative evaluation of the different parameters could be of relevant interest for discriminating the different effect of composition (nanofiller) and processing (draw ratio). Various parameters have been calculated from tensile mechanical properties, according to Equations (3)–(6) and (9)–(11). Draw Stiffening Factor, Efficacy of Drawing and Efficacy of Filler are summarized in Table 4.

Draw Stiffening Factor (DSF) is calculated for each composition as the ratio of modulus of drawn fiber (*E*_drawn_) as function of modulus of undrawn fiber (*E*_DR1_)

(9) $$\mathrm{DSF}=\frac{E_{\mathrm{drawn}}}{E_{\mathrm{DR}1}}$$

The Efficacy of Drawing (DE) for each composition is evaluated as the ratio between the draw stiffening factor DSF and the correspondent draw ratio

(10) $$\mathrm{DE}=\frac{\mathrm{DSF}}{\mathrm{DR}}$$

The Efficacy of Filler (FE) for each composition is evaluated from the difference of nanocomposite modulus (*E*_NC_) and the modulus of PP normalized to the volume fraction of the filler (*f*) and to the modulus of PP (*E*_PP_)

(11) $$\mathrm{FE}=\frac{E_{\mathrm{NC}}-E_{\mathrm{PP}}}{f\;E_{\mathrm{PP}}}$$

The stiffening effect can be visualized from the relative tenacity and the relative modulus at various draw ratio. For instance, the DSF and DE indicates the stiffening effect of 0.25 vol % and 0.5 vol % of filler for DR10 and DR15, whereas some limitation in drawing could be deducted for nanocomposite fibers AR974-1 and AR974-2. The consistent effect of filler on fiber properties appears evident from the parameter FE, filler efficiency, for composition 0.25 and 0.5 vol % of silica at all the draw ratios, especially for DR10. From these findings, it is clear in general that for the production of nanofilled fibers the maximum draw ratio should be requested, but the filler content should be properly defined.

Factorial regression of experimental trials of Series II (Table S8, Supplementary Materials) has been performed versus draw ratio and fumed silica in terms of analysis of variance, coded coefficients, regression equation and fits/diagnostics for tensile modulus (Tables S9–S11, Supplementary Materials), for tenacity (Tables S12–S14, Supplementary Materials), and for stress at break (Tables S15–S17, Supplementary Materials), respectively. The role of the two factors, draw ratio and fumed silica has been evaluated with both linear terms and at two-way interactions. According to this analysis of Series II data, the term Draw Ratio was effective on mechanical properties with probability value more than 99%, being *p*-value = 0.000, as shown in the Table 5 for tensile modulus, tenacity and stress at break. It is worth noting that the term AR974 is below the threshold of effective significance, according to the chosen level of risk α = 0.05. In fact, *p*-values 0.089, 0.065 and 0.0100 were determined in the analysis of variance of tensile modulus, tenacity and stress at break, respectively. Moreover, in all the cases, the interaction among the two terms, draw ratio and fumed silica, is absolutely negligible, with *p*-values in the range 0.17–0.49.

**Figure 6:**
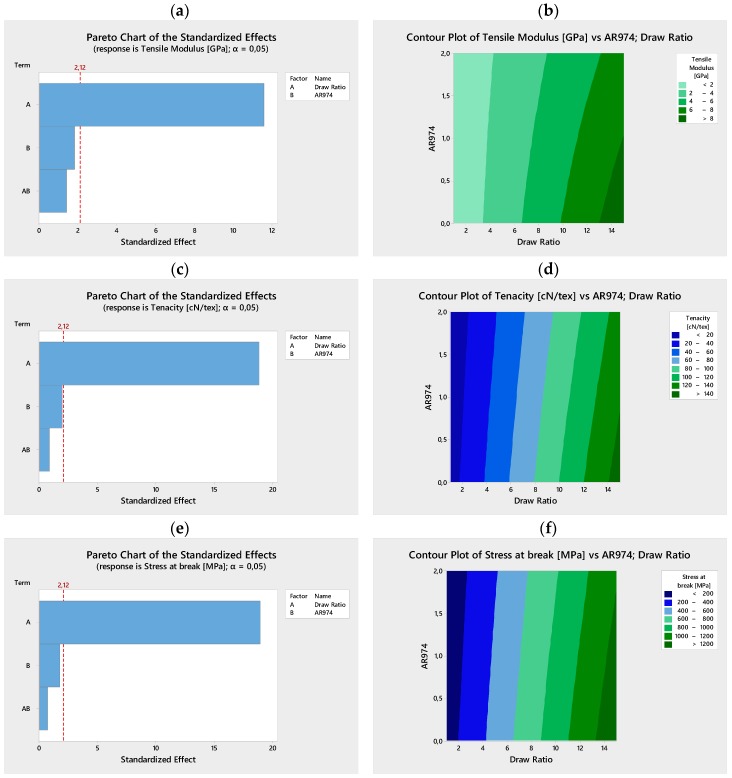
Series II. Pareto charts (**a**,**c**,**e**) and Contour plots (**b**,**d**,**f**) of the standardized effects on tensile modulus, tenacity and stress at break. The significance of draw ratio is confirmed as main factor.

Pareto charts in Figure 6a,c,e well summarize the statistical analysis of selected mechanical data reported in Table 3. The contour plots in Figure 6b,d,f evidence the main role of drawing in improving mechanical properties (tensile modulus, tenacity and stress at break) of nanocomposite fibers. Moreover, it is possible to visualize that the higher the filler content, the higher the drawing should be to achieve analogous improvement of mechanical properties. The slope and the linearity of the “vertical” curves in Figure 6b decrease with the draw ratio, and suggest a progressive deviation that could be attributed to the negative effect of the filler. Moreover, the plot of tensile modulus could be related to parametric evaluation of the Filler Efficiency (FE) reported in Table 4. In particular, the higher the nanofiller content, the lower the FE at constant draw ratio; and the higher the draw ratio, the more pronounced the decrease of Filler Efficiency with the filler content (the negative value of FE for nanofibers at composition of 2 vol % AR974 and DR10 or DR 15 is noteworthy).

Supplementary Material shows more details on the factorial analysis of tensile modulus, tenacity and stress at break. In particular, interaction plot, normal plot of the standardized effects, as well as compared plots of the main effects on tensile modulus (Figures S5–S7, Supplementary Materials), tenacity (Figures S8–S10, Supplementary Materials), and stress at break (Figures S11–S13, Supplementary Materials), are presented.

According to these analyses on Series II, a positive effect on mechanical properties could be evaluated for the draw ratio only.

### 3.3. Short-Term Creep Tests

**Figure 7:**
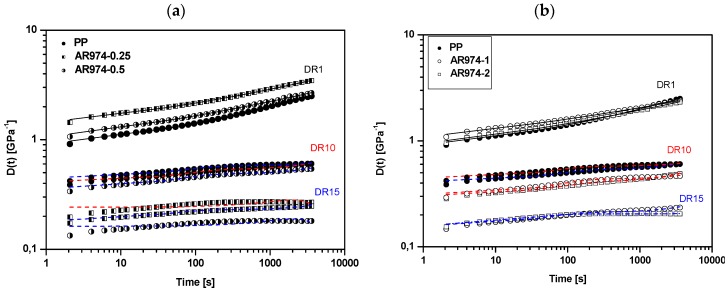
Tensile compliance of neat and nanocomposite PP fibers. Silica content: (**a**) 0.25% or 0.50% by vol; (**b**) 1% or 2% by vol. Draw ratios DR = 1 (black line), DR = 10 (red line), DR = 15 (blue line). The fitting lines follow the Burgers model (see the following).

Many practical applications of composite fibers encompass long-lasting applied loads, which make creep analysis and modeling inevitable [47]. In our simplified experiments, the strain of PP/fumed silica fibers was monitored as a function of time at a constant stress of 3 MPa applied for 3600 s. Creep compliance curves of neat and nanofilled fibers with different draw ratios are reported in Figure 7a,b. For as-spun fibers, the creep compliance of the fibers with 0.25–0.5 vol % of nanofiller is higher by about 30%–50% than that obtained for neat PP, while for the compositions with 1 and 2 vol % no significant variation of creep compliance was observed.

On the other hand, the incorporation of fumed silica contributes to remarkable reduction of the creep compliance of drawn specimens. The largest decrease in compliance is achieved for compositions with nanofiller fractions 0.25 and 0.5 vol %, in agreement with tensile modulus (see Table 3). Similar results were reported for nanosilica composites [27,48,49] with HDPE matrix or PP matrix [50]. Lower creep compliance of the fibers with higher nanofiller fractions and DR15 might partly be related to a higher fraction of immobilized matrix entrapped in the agglomerates of nanofiller particles. The higher creep resistance of nanocomposite fiber was manifested by composition AR974-0.25 and AR974-0.5 at DR10 or DR15.

To model the creep behavior of the investigated fibers, the Burgers model, i.e., a four-elements mechanical model composed of a series combination of the Maxwell and Kelvin models, has been adopted [51]. In terms of the Burgers model, the creep compliance can be expressed as:

(12) $$D\left(t\right)=\frac{1}{E_{M}}+\frac{t}{\eta_{M}}\;+\;\frac{1}{E_{K}}\;\left[1\;‒\;exp\left(\frac{E_{K}\;t\;}{\eta_{K}}\right)\right]$$

where *E*_M_ and η_M_ are elastic and viscous components of the Maxwell sub-model, and *E*_K_, η_K_ are analogous components of the Kelvin sub-model. The outcome of the Burgers model is represented with best fitting lines in Figure 7.

It can be observed that the Burgers model can effectively predict the creep compliance of both neat PP and nanocomposite fibers. Examining the values of the as-spun nanocomposite fiber reported in Table 6, an almost linear dependence of all fitting parameters on the filler content for composition 0.25–1 vol % of fumed silica can be observed. Moreover, as the draw ratio increases, an enhancement of elastic and viscous components can be noticed, which is in conformity with data reported for polyamide fibers [52]. It is well evident that both elastic (*E*_K_, *E*_M_) and viscous (η_K_, η_M_) parameters of all drawn fibers are higher than those of the as-spun fiber, but some differences can be noticed in dependence on the filler content. In particular, the compositions with 0.25 and 0.5 vol % of nanofiller exhibited the higher elastic E_M_ parameter (about 5–7 GPa) in conformity with the higher tensile modulus, as reported in Table 3, and a high creep resistance could be obtained for both DR10 and DR15. On the other hand, according to fitting parameter the high creep resistance of AR974-1 and AR974-2 fibers drawn at DR15 can be mainly attributed to the viscous parameter (η_K_ = 95–105 TPa·s). We can conclude that the creep resistance of PP has been significantly enhanced by the fumed silica addition, especially for drawn fibers with low nanofiller fractions (0.25–0.5 vol %). At higher fumed silica content, analogous creep resistance could be achieved at high draw ratio only (DR ≥ 15).

### 3.4. Thermal Properties of Composite Fibers

**Figure 8:**
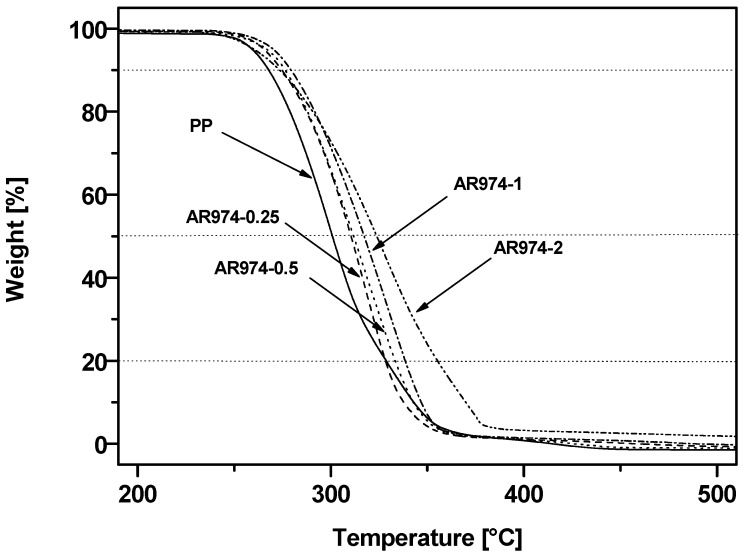
Thermogravimetric analysis (TGA) thermograms of the neat PP and of the PP matrix in the as-spun fibers with various nanofiller fractions.

Beneficial effect of fumed silica on the thermal degradation resistance of all composites (with respect to the neat PP) is documented by Figure 8.

It is instructive to compare the thermal stability of the investigated materials at selected decomposition temperatures (Table 7). The temperatures *T*_0.1_, *T*_0.5_ and *T*_0.8_ corresponding to the mass losses of 10%, 50% and 80% (for selected heating rate) of the PP-fumed silica fibers are higher than those of the neat PP, which confirms expected stabilizing effect of nanofiller particles under oxidizing atmosphere even at very low silica fractions. Analogous improvement has been reported for PP fibers containing fumed silica [14] or polyethylene plates with fumed silica or hydrotalcite [27,40,46]. Improved thermo-oxidative stability, manifested by the shifts of *T*_0.1_, *T*_0.5_ and *T*_0.8_ toward higher temperatures, can be ascribed to the barrier effect of the nanoparticles hampering the diffusion of the gaseous degradation products [53]. *T*_0.1_ for the composite with 2 vol % of AR974, which is slightly lower than the corresponding temperature of other PP-silica nanofibers, can tentatively be related to possible aggregate formation accounting for less effective barriers to diffusion. On the other hand, the effect of addition of 2 vol % fumed silica is evident at higher degradation level (higher temperatures of decomposition *T*_0.5_ and *T*_0.8_) where silica nanoparticles create a temporary protection barrier. It is also worth noting that the peak of the derivative curve of TGA reveals the tendency not only to shift the peak towards higher temperature, but also to reduce the maximum degradation rate, proportionally to the addition of fumed silica (see Table 7).

### 3.5. Microstructural Characterization

**Figure 9:**
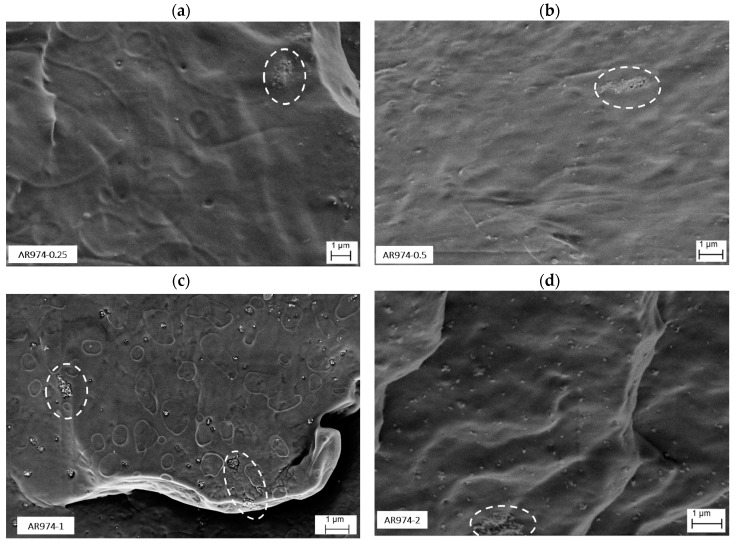
SEM images of cross-section view of as-spun PP nanocomposite fibers (DR1) with different nanosilica content 0.25 vol % (**a**); 0.5 vol % (**b**); 1 vol % (**c**); and 2 vol % (**d**). Circles evidence silica aggregates.

SEM images of as-spun nanocomposite fibers are reported in Figure 9a–d.

For low nanofiller contents of 0.25 and 0.5 vol % (Figure 9a,b), well-dispersed silica nanoparticles are visible along with relatively small agglomerates of an average size in the range of about 100–200 nm, even though a few larger agglomerates can be evidenced in the dot zones in the figures. As the silica fraction increases, larger agglomerates of the filler appear. Agglomerates up to 500–800 nm can be observed for the filler fraction content of 1 vol % (Figure 9c) and 2 vol % (Figure 9d). These results are in conformity with previous research where similar sizes of nanosilica aggregates of particles were observed [22,25,34]. The aggregated morphology, observed for compositions with higher silica fractions, can be attributed to the strong interaction between the nanoparticles which becomes more and more important as the particle concentration increases [27].

**Figure 10:**
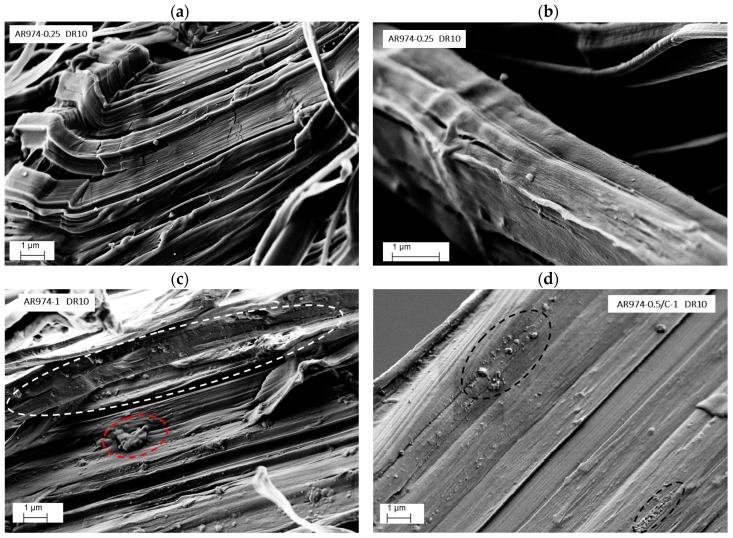
SEM images of transversal view of drawn PP nanocomposite fibers (DR10) with different nanosilica content 0.25 vol % (**a**,**b**); 1 vol % (**c**); and 0.5 vol % with compatibilizer 1% vol % (**d**). Circles evidence silica aggregates.

Drawn fibers cooled in liquid nitrogen could not be fractured transversally, due to the higher tenacity, but they broke longitudinally along the main axis, as shown in Figure 10. Multifibrillar structure of fiber with 0.25 vol % of AR974 (Figure 10a) can be observed with the presence of mainly small fumed silica particles with a dimension up to about 200 nm. On the other hand, at higher filler content, some large aggregates are still present in nanocomposite fiber with 1 vol % of AR974 and drawn ten times, as shown in Figure 10c (see the red dot zone); even though many of the particles have been aligned along the fiber after disintegration or partial disintegration of aggregates during the drawing process (see the white dot zone).

Quite analogous distribution of nanofiller particles in drawn fiber containing the compatibilizer is evidenced by Figure 10d. Thus, any improvement in fumed silica dispersion cannot be observed, but the compatibilizer seems to account for smoother and regular fracture surface.

**Figure 11:**
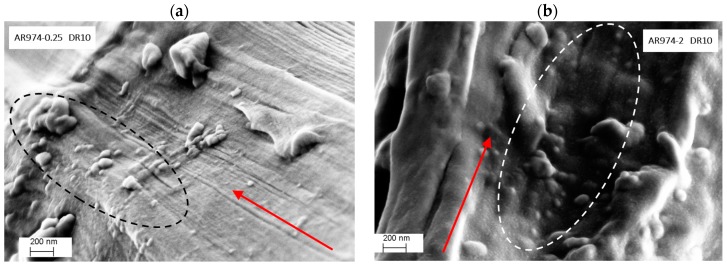
High magnification SEM images of transversal view of drawn PP nanocomposite fibers (DR10) with nanosilica content of 0.25 vol % (**a**); and 2 vol % (**b**). The arrow indicates the fiber orientation; the circles evidence some fumed silica particles.

Higher magnification of longitudinal fracture surface of nanocomposite fibers without compatibilizer makes it possible to confirm (i) the better dispersion of nanoparticles in drawn fiber containing 0.25 vol % of fumed silica (Figure 11a) with respect to that with 2 vol % of AR974 (Figure 11b); (ii) higher orientation of polymer fibrils in nanocomposite at lower nanofiller content, which accounts for the higher stiffness and tenacity.

These observations allow us to conclude that various fumed silica particles have been aligned along the fiber axis during drawing, and that agglomerate size is inversely proportional to fiber drawing. The higher the filler content, the higher the draw ratio at which the disintegration of aggregates can be expected.

### 3.6. X-ray Diffraction (XRD) Analysis of the PP and Nanocomposite Fibers

**Figure 12:**
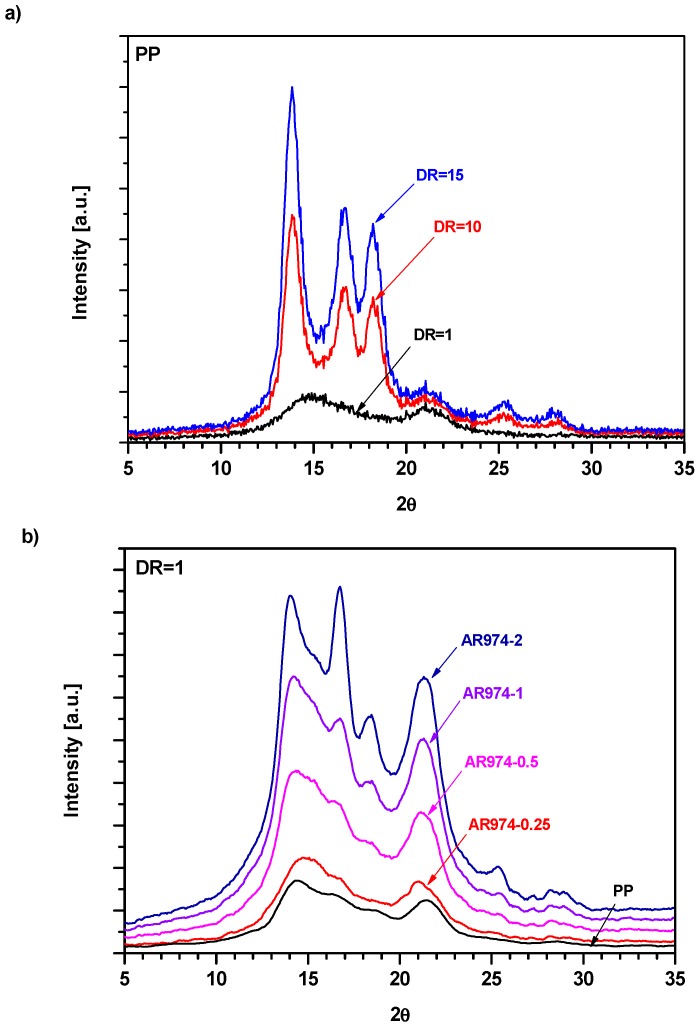

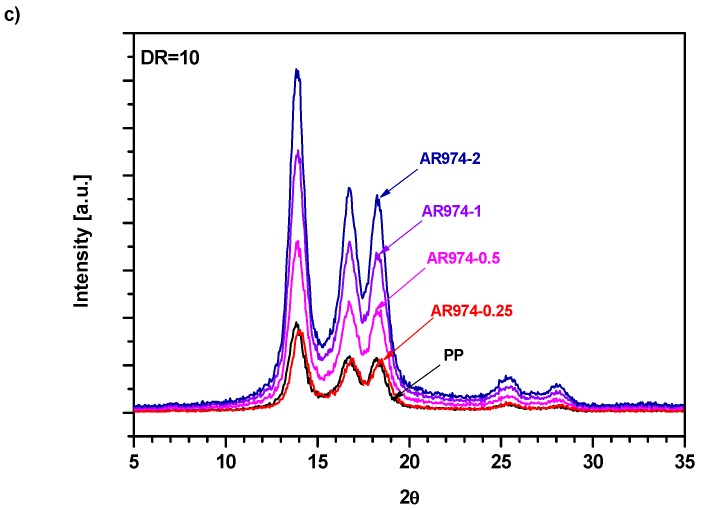
(**a**) Effect of draw ratio on XRD pattern of neat PP and effect of fumed silica content on XRD patterns of nanocomposite at (**b**) DR = 1 and (**c**) DR = 10.

XRD spectra of neat and nanofilled PP fibers are reported in Figure 12. As-spun PP fiber (DR = 1) is characterized (Figure 12a) by two broad peaks centered at 2θ values of about 14.8° and 21° in 2θ [54,55]. They could be attributed to a mesomorphic form of isotactic PP characterized by not well-defined crystalline structures [56]. Drawn fibers (DR10 or DR15) show well-defined and more intense peaks. Incorporation of fumed silica into fibers (DR1) accounts for marked modifications of the observed XRD patterns (Figure 12b). Combined effects of silica fraction and drawing are visualized in Figure 14c. For the compositions with higher fractions of fumed silica (Figure 12b,c), XRD patterns clearly display—along with the broad peak centered at 21°—up to five distinct peaks at 2θ values of about 14°, 17°, 18.5°, 25.5° and 28°, which can be associated with an isotactic α-polypropylene crystalline phase according to Powder Diffraction File card n. 50-2397 [57].

Figure 12 reveals several important features: (i) increasing of drawing leads to progressive increase in the peak of the α-crystalline phase and the reduction of the peak related to mesomorphic phase; (ii) the previous peaks can be attributed to the corresponding reflexes, (110), (040), (130), (060) and (220), respectively; (iii) comparing the experimental patterns with the standard phase of PP [55], it is noteworthy that the absence of the (111) and (−131) reflexes of the α-phase, moreover, (iv) an inversion between the relative intensities of (040) and (130) reflexes can be noticed, with respect to the above-reported neat phase.

Crystallinity data, evaluated by a deconvolution process [40] of the amorphous and α-crystalline phases evidence that fumed silica leads to slightly higher crystallinity content: *X*c = 24% for neat PP and about 28% for AR974-2 sample (Table 8).

Relative crystallinity fractions expressed in terms of the intensity ratios for a selected peak [58] are reported in Table 8. The ratio *I*(040) for each nanocomposite sample over *I*(040)ref for the as-spun (DR1) neat polypropylene fiber, set as reference material, shows an increase for all compositions with a silica fraction (Table 8). This allows us to conclude that fumed silica in polypropylene matrix may act as a nucleating agent [14,16]. A comparable trend is also observed for the *I*(130)/*I*(130)ref ratio, even if the drawing effect seems to be more important, as discussed in the following.

For as-spun material, the presence of two large peaks on a broadened ground suggests the coexistence of both the crystalline and amorphous phases. After the drawing process, a new sharp peak appears which can be attributed to the development of α-crystal in PP [58,59]. If the crystallinity percentage is compared, it is evident that (i) hot drawing improves crystallization, as usual in fiber orientation; (ii) the crystalline fraction increases from 24% for as-spun PP up to 58% for DR = 10; and (iii) with increasing DR up to 15, any further increase in crystallinity is not observed (*X*c = 56%). The same trends are observed for all studied PP nanofilled fibers. As summarized in Table 8, the highest values of crystallinity were obtained for fibers drawn to DR10, while at DR15 the crystallinity is somewhat lower. The crystallite size estimated by means of the Scherrer equation from the data for the three more intense peaks of the α-phase (110), (040) and (130) reflections are summarized in Table 9.

In the as-spun fibers, very small crystallites are found, but their dimensions rise with the drawing process (up to DR = 10), which is in conformity with previous observations [60]. At draw ratio DR15, the tendency of a slight reduction of crystal size might be interpreted as a direct consequence of the decrease in crystallinity (see Table 8).

It can be concluded that the addition of the nanofiller leads to a more ordered structure as the detected peaks become sharper and more distinct (Figure 12b), thus confirming the presence of the α-crystalline phase of isotactic PP. This finding is a clear indication that fumed silica acts as a nucleating agent for the PP matrix. For the as-spun fibers (DR1), the nanofiller addition seems to play a key role because more crystallized structures can be obtained by increasing the silica nanoparticle content. This trend is documented by evaluated parameters, mainly intensity ratios and crystallite sizes (Table 9). Drawing process accounts for increase in the crystallite dimensions; on the other hand, the effect of nanofiller becomes less significant because the crystallization process is dominated by higher molecular orientation [61]. It can be concluded that the effect of nanofiller on fiber structure is more remarkable at low drawing ratios where the filler acts as a nucleating agent. With higher drawing ratios, the role of nanofiller is less significant because the crystallization process is dominated by molecular orientation during drawing [61].

### 3.7. Dynamic Mechanical Thermal Analysis (DMTA)

**Figure 13:**
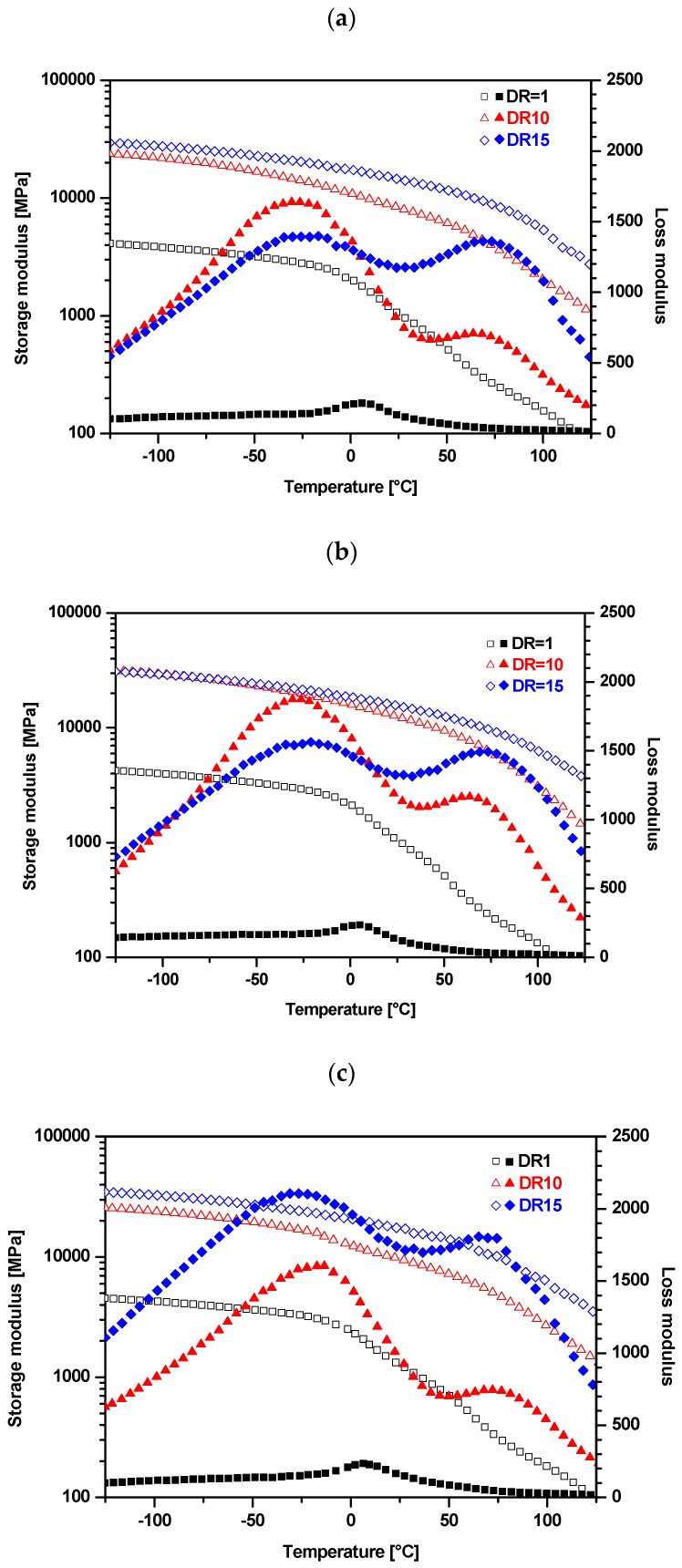
Tensile storage modulus (empty symbols) and loss modulus (full symbols) of neat and selected nanofilled PP fibers at various draw ratios. (**a**) PP; (**b**) AR974-0.5; and (**c**) AR974-1.

Dynamic mechanical thermal analysis has been used for evaluating the tensile storage *E*′ and loss moduli *E*” of composite fibers (Figure 13). Generally, the interaction between nanoparticles and polymer matrix is expected to restrict the mobility of polymer segments adjacent to the particle surface. Consequently, the sub-glass transitions and the glass transition of the matrix may be shifted towards higher temperatures [45]. The effects of (i) silica fraction and (ii) DR on *E*′ of composite fibers are summarized in Table 10. As can be seen, *E*′ of the as-received (DR1) composite fibers only slightly rises with the silica fraction. On the other hand, *E*′ of the composite fibers markedly rises with the drawing ratio up to DR15 (Figure 13), but the highest values of *E*′ are reached for the compositions with the nanofiller fractions 0.25 and 0.5 vol % at DR10 and DR15. Loss modulus *E*” dependences on temperature (Figure 13) reveal that neat PP for DR1 exhibits a small loss peak (generally designated as the β relaxation) located at about 5 °C (Figure 13a), which corresponds to the glass transition of undrawn PP with mesomorphic morphology. Incorporation of 0.5 and 1 vol % of nanosilica (Figure 13b,c) to undrawn PP does not visibly affect the size and temperature location of the β peak, as shown in literature [34,61]. In contrast, XRD patters show (Figure 13b) pronounced changes indicating partial transformation of the original mesomorphic form of PP into α-crystalline form. Therefore, it seems that DMTA analysis is not enough sensitive to indicate neither these morphological changes, nor possible immobilization of thin layers of the PP matrix adjacent to filler surface.

On the other hand, XRD as well as DMTA patterns are markedly affected by drawing procedure (Figure 12 and Figure 13). PP specimens with DR10 and DR15 show two pronounced loss peaks: β peak at about −28 °C and α peak at about 75 °C. As can be seen, the α relaxation is related to DR, but it is not induced by the present nanofiller at DR1 (in other words, there is no indication of the presence of interphase layer with reduced molecular mobility in undrawn composite fibers). The intensity (the height of a loss peak) of the glass transition β peak of the neat PP markedly increases with DR, whereby *T*_β_ shifts to lower temperatures (Table 11), which is in conformity with our previous observation [15]. The α peak (or α relaxation [62]) observed at DR10 and DR15 (but not at DR1) has to be attributed to more hindered molecular motions, whose onset requires higher temperatures (energies) than the β motion in amorphous regions. It seems obvious that the α-relaxation is associated with limited molecular motions (hindered rotations) in the α-crystalline regions formed during drawing (as documented by Figure 12). The intensity of the β and α relaxations of drawn fibers evidences their tendency to increase with the nanofiller fraction. The effect of the filler may be amplified proportionally to DR, because destruction of the filler aggregates (and increase of the contact area matrix/filler) by shear forces in the course of drawing is proportional to draw ratio.

We can attempt to better explain the shifts of *T*_β_ and *T*_α_ induced by nanofiller fraction and/or drawing DR by correlating DMTA and XRD data (Figure 12 and Figure 13). Neat PP at DR1 shows only one loss maximum—very much like amorphous polymers—which can be identified with glass transition of amorphous regions in the mesomorphic PP matrix (in this paper we will not consider the sub-glass transitions, if any). As can be seen, temperature location at about 5 °C and the size of the β peak of PP are not perceptibly affected by incorporated silica. The α peak located at about 70 °C is exclusively exhibited by drawn specimens, regardless of the fraction of silica. Increasing DR accounts for decrease in *T*_β_ (from about 5 to −25 °C), while *T*_α_ remains located around 74 °C. XRD reveals (Figure 12) that drawing causes the transformation of the mesophase (“one-phase structure”) of undrawn specimens into two separated phases, i.e., amorphous and α-crystalline phases. Temperature *T*_β_ = −25 °C of drawn specimens corresponds to standard *T*g given for isotactic PP.

Figure 13 also shows that for the neat PP and composite with 0.5% of silica the height of the α-loss maximum rises when DR rises from 10 to 15, which is accompanied by reduction of the β loss maximum (temperature dependences of *E*” are crossing at about 10 °C). This “transformation” of the loss peaks is the manifestation of the increasing fraction of the α-crystalline phase (due to drawing) and, consequently, of the decreasing fraction of amorphous phase.

Table 11 evidences that the α-loss peak rises with nanoparticle fraction and draw ratio. Besides, a shift towards higher temperatures can be seen, i.e., from 67–69 °C for neat PP up to 73–76 °C for fibers of AR974-1 drawn to DR10 and for fibers of AR974-2 drawn to DR15. These data indicate that the drawing process accounts for improving the dispersion of the nanoparticles in the matrix and formation of a more compact arrangement of chain segments.

**Figure 14:**
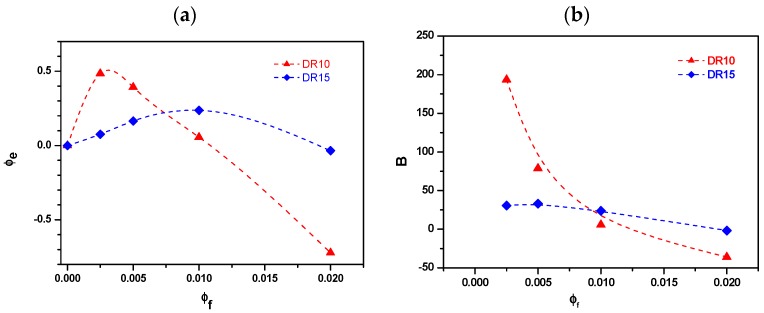
(**a**) Effective particulate volume fraction (ф_e_); and (**b**) effective particulate volume per single particle (B) of neat and nanofilled PP fibers as a function of silica volume fraction, according to the Sumita model [63].

To quantitatively characterize the interphase thickness, the effective particle volume fraction (ф_e_) and effective volume per single particle (B-parameter) were estimated according to Sumita’s model [63] through Equation (13):

(13) $$\frac{E{”}_{c}}{E{”}_{p}}={\left(1-\phi_{e}\right)}^{-1}={\left(1‒\phi_{f}B\right)}^{-1}$$

where *E*_c_” and *E*_p_” represent the maximal loss moduli of the composites and the neat polymer in DMTA measurements. The ratio *E*_c_”/*E*_p_” is calculated by using the α-relaxation peak height values (Table 11); the obtained ф_e_ and B values are plotted in Figure 14 as function of the fumed silica fraction (ф_f_). As evidenced by Figure 14a, the effective volume ф_e_ of the material drawn with DR10 increases up to 0.25 vol % of the nanofiller and then slowly decreases with silica fraction.

The same trend can be observed for DR15; however, in this case, the maximum of effective volume (0.25) is reached for the composition with 1 vol % of fumed silica. The effective volume per single particle (B parameter in Figure 14b) shows a trend similar to that of ф_e_. This means that the thickness of the PP layer immobilized on the surface of fumed silica nanoparticles is limited, due to the presence of agglomerates. Additionally, the decreasing B-parameter suggests that the extent of the particle agglomeration increases with the filler content [15,63]. A similar trend was already observed for HDPE fibers with hydrotalcite [64]. In fact, thin interlayer and low nanofiller content are beneficial for simultaneous improvement of interfacial adhesions and dispersion of the nanoparticles.

The Sumita model was applied to tentatively evaluate the effective volume fraction of the immobilized phase adjacent to filler surface by using data on loss modulus peak. The relative maximum matrix fraction immobilized on the filler surface was observed for 0.25 and 1.0% vol. of nanofiller, suggesting a good dispersion of nanosilica in drawn fibers.

## 4. Conclusions

This study simultaneously describes the effect of the nanofiller content in the nanocomposite fiber and the effect of fiber drawing.

Production of nanocomposite fibers/filaments consisting of isotactic polypropylene matrix and surface-treated fumed silica AR974 was realized via the double step process consisting of melt extrusion and drawing. As-spun fiber could be easily obtained after compounding by means of twin-screw extrusion for composition in between 0.25% and 2% by volume of nanofiller.

Scanning electron microscopy (SEM) microphotographs show that for low nanofiller contents (0.25 vol % and 0.5 vol %) well-dispersed silica nanoparticles are visible along with relatively small agglomerates (average sizes in range of about 50–100 nm). The size of agglomerates in as-spun fiber increases with the filler fraction and achieves 500–800 nm for 2 vol % of the filler. Partial disintegration of agglomerates and alignment of particles along the fiber axis has been evidenced after drawing.

X-ray diffraction (XRD) analysis of the polypropylene (PP) crystallinity evidences that fumed silica induces formation of a slightly higher crystalline fraction: *X*c = 24% for neat PP, while about *X*c = 28% was found for the sample with 2 vol % of AR974 silica. The increase of dimensions of crystallite size from about 3 nm determined for neat polypropylene, to about 5–12 nm found for all nanocomposite compositions, allows us to conclude that fumed silica in polypropylene matrix acts as a moderate nucleating agent.

As for the mechanical properties of nanofilled fibers, tensile modulus and tensile stress at break rose with (i) the silica fraction merely up to 1 vol %; and with (ii) increasing draw ratio of all samples throughout the draw ratio (DR) interval tested. Various parameters have been proposed for the evaluation of drawing effect and the filler content. In particular, the Efficacy of Filler put in evidence the relevant effect of 0.25 vol % and 0.5 vol % of fumed silica in both as-spun and drawn fibers.

Simplified tensile creep tests of as-spun fibers showed that the tensile compliance of the fibers with 0.25–0.5 vol % of nanofiller is lower by about 30%–50% than that of neat PP, while for the compositions with 1 and 2 vol % no significant variation of creep compliance was observed. Analogous creep tests evidenced the reduction of creep compliance—with respect to the neat PP fibers—over the whole range of investigated draw ratios.

Also the storage modulus *E*′ and loss modulus *E*” from the dynamic mechanical thermal analysis (DMTA) tensile tests confirmed the stiffening effect of fumed silica in PP composites. Similar to static tensile tests, also *E*′ was found to rise with the draw ratio of test fibers. A relative maximum at room temperature of *E*′ = 15.8 GPa was found for the composition with 0.5 vol % of nanofiller and DR15. The maximum values *E*” of 2.1 GPa (β-peak) and 1.8 GPa (α-peak) were achieved for 1.0 vol % and DR15.

The incorporation of the nanofiller in the PP matrix also enhanced the thermal stability of composite fibers in comparison to neat PP as manifested by shifting the temperature of the maximum degradation rate from about 300 to 330 °C for composition with 1–2 vol % of nanofiller.

The results confirm our previous data that polypropylene effectively reinforced with 0.25–2 vol % of hydrophobic fumed silica surface modified either with octylsilane (AR805) or with dimethyldichlorosilane (AR974), can be easily spun and also drawn into nanofilled fibers with tenacity up to 137 cN/tex.

Sumita model was applied to tentatively evaluate the effective volume fraction of the immobilized phase adjacent to filler surface by using data on loss modulus peak. The relative maximum matrix fraction immobilized on the filler surface was observed for 0.25 and 1.0 vol % of nanofiller, suggesting a good dispersion of nanosilica in draw fibers.

Following statistical analysis in DOE performed by Minitab 17 software, the two series evidenced the significant effect of draw ratio on fiber properties.

Surprisingly enough, the effect of added compatibilizer in this type of nanocomposites (fumed silica in PP; fiber spinning and drawing) was found insignificant. In the same time, according to Series I evaluation, the contribute of fumed silica AR974 nanofiller is significantly relevant at 0.5% by vol.

The compositions with 0.5 vol % of fumed silica at various draw ratio, and with 1 vol % at high draw ratio are found to be the most promising for low-cost improvements of mechanical properties and thermal resistance of produced fibers.

## Figures and Tables

**Table 1:** Designation and composition of polypropylene (PP) nanocomposites fibers.

Fiber	Fumed silica (vol %)	PP (vol %)	PP-*g*-MA (vol %)	MFI (g/10 min)
PP	0	100	0	1.84 ± 0.08
AR974-0.25	0.25	99.75	0	2.04 ± 0.10
AR974-0.5	0.5	99.50	0	2.40 ± 0.14
AR974-1	1	99.0	0	2.52 ± 0.08
AR974-2	2	98.0	0	2.69 ± 0.20
AR974-0.5/C-0.5	0.5	99.0	0.5	3.20 ± 0.15
AR974-0.5/C-1	0.5	98.5	1.0	3.62 ± 0.20

**Table 2:** Analysis of variance of tensile modulus (Series I) versus draw ratio, fumed silica (AR974) and compatibilizer (C).

Source	DF	Adj SS	Adj MS	*F*-Value	*p*-Value
Model	7	501.127	71.590	95.29	0.000
Linear	3	465.500	155.167	206.54	0.000
Draw Ratio	1	439.247	439.247	584.67	0.000
AR974	1	7.206	7.206	9.59	0.004
C	1	0.451	0.451	0.60	0.443
2-Way Interactions	3	9.689	3.230	34.30	0.010
Draw Ratio x AR974	1	2.835	2.835	3.77	0.059
Draw Ratio x C	1	0.436	0.436	0.58	0.451
AR974 x C	1	4.336	4.336	5.77	0.021
3-Way Interactions	1	0.703	0.703	0.94	0.339
Draw Ratio x AR974 x C	1	0.703	0.703	0.94	0.339
Error	40	30.051	0.751		
Total	47	531.178			

**Table 3:** Titer, tenacity and other mechanical properties of neat and nanofilled PP fibers at selected draw ratios (DR).

Draw ratio	Fiber	Titer * (tex)	Tenacity (cN/tex)	Diameter (µm)	Tensile modulus (GPa)	Stress at break (MPa)	Strain at break (%)
DR1	PP	174 ± 2	9.2 ± 0.4	495 ± 6	0.48 ± 0.01	83 ± 4	1260 ± 15
	AR974-0.25	182 ± 3	8.2 ± 0.3	506 ± 9	0.58 ± 0.04	74 ± 3	1160 ± 45
	AR974-0.5	179 ± 7	7.7 ± 0.7	500 ± 20	0.68 ± 0.02	70 ± 6	1144 ± 54
	AR974-1	180 ± 3	9.1 ± 0.7	500 ± 8	0.65 ± 0.01	83 ± 6	1282 ± 35
	AR974-2	186 ± 3	6.7 ± 0.9	506 ± 9	0.67 ± 0.02	62 ± 8	1090 ± 58
	AR974-0.5/C-0.5	181 ± 1	7.7 ± 0.7	503 ± 4	0.60 ± 0.02	70 ± 6	1304 ± 80
	AR974-0.5/C-1	184 ± 3	7.7 ± 0.2	507 ± 8	0.66 ± 0.04	70 ± 2	1251 ± 42
DR5	PP	37.6 ± 0.5	41.3 ± 1.4	230 ± 3	1.71 ± 0.15	374 ± 13	185 ± 11
	AR974-0.25	37.7 ± 0.7	55.4 ± 1.8	230 ± 4	2.45 ± 0.11	503 ± 16	117 ± 5
	AR974-0.5	36.2 ± 0.8	40.5 ± 1.1	225 ± 5	2.62 ± 0.17	369 ± 10	109 ± 10
	AR974-1	35.1 ± 0.3	48.3 ± 2.7	221 ± 2	2.41 ± 0.13	442 ± 25	71 ± 14
	AR974-2	36.8 ± 0.7	37.3 ± 1.8	225 ± 4	1.81 ± 0.16	346 ± 17	129 ± 13
	AR974-0.5/C-0.5	37.8 ± 0.5	40.6 ± 1.6	230 ± 3	1.76 ± 0.11	370 ± 15	162 ± 10
	AR974-0.5/C-1	35.5 ± 0.5	53.3 ± 2.2	223 ± 1	2.44 ± 0.17	485 ± 20	96 ± 15
DR10	PP	17.7 ± 0.3	104.3 ± 2.8	158 ± 3	5.30 ± 0.15	944 ± 25	53 ± 5
	AR974-0.25	17.1 ± 0.4	127.0 ± 3.3	155 ± 4	7.50 ± 0.20	1153 ± 30	46 ± 2
	AR974-0.5	17.2 ± 0.2	115.8 ± 4.9	155 ± 2	8.62 ± 0.55	1054 ± 45	43 ± 6
	AR974-1	17.3 ± 0.5	99.9 ± 4.9	155 ± 4	5.70 ± 0.45	915 ± 45	36 ± 12
	AR974-2	17.5 ± 0.6	85.8 ± 1.9	155 ± 5	4.10 ± 0.35	795 ± 18	51 ± 10
	AR974-0.5/C-0.5	17.2 ± 0.1	107.2 ± 2.2	155 ± 1	5.80 ± 0.30	976 ± 20	33 ± 13
	AR974-0.5/C-1	17.2 ± 0.3	111.5 ± 3.8	155 ± 3	7.04 ± 0.25	1015 ± 35	35 ± 10
DR15	PP	11.6 ± 0.2	137.0 ± 5.5	128 ± 2	7.88 ± 0.35	1240 ± 50	34 ± 3
	AR974-0.25	11.3 ± 0.2	135.5 ± 1.4	126 ± 2	8.30 ± 0.50	1230 ± 13	36 ± 4
	AR974-0.5	11.3 ± 0.4	135.9 ± 2.7	126 ± 4	9.41 ± 0.25	1237 ± 25	28 ± 6
	AR974-1	10.9 ± 0.2	137.1 ± 5.5	123 ± 2	8.10 ± 0.35	1256 ± 50	32 ± 8
	AR974-2	11.9 ± 0.3	123.6 ± 4.1	128 ± 3	6.50 ± 0.40	1145 ± 38	37 ± 5
	AR974-0.5/C-0.5	11.2 ± 0.5	133.0 ± 2.2	125 ± 5	9.26 ± 0.18	1211 ± 20	30 ± 7
	AR974-0.5/C-1 **	11.5 ± 0.3	119.7 ± 3.0	127 ± 3	8.92 ± 0.20	1118 ± 27	27 ± 6

* for definition of Linear density and Tenacity see ASTM D861 Standard Practice for Use of the Tex System to Designate Linear Density of Fibers, Yarn; ** Fiber drawn at DR = 12 (see Figure 1).

**Table 4:** Relative tenacity (RT) and relative tensile modulus (RTM) at constant draw ratio or as function of undrawn PP fiber. Draw Stiffening Factor (DSF), Drawing Efficacy (DE) and Filler Efficiency (FE).

Draw ratio	Fiber	RT_DR_ Equation (3)	RT_PP_ Equation (5)	RTM_DR_ Equation (4)	RTM_PP_ Equation (6)	DSF Equation (9)	DE Equation (10)	FE Equation (11)
DR1	PP	1.00	1.00	1.00	1.00	1.00	1.00	n.d.
	AR974-0.25	0.89	0.89	1.21	1.21	1.00	1.00	83
	AR974-0.5	0.84	0.84	1.42	1.42	1.00	1.00	83
	AR974-1	0.99	0.99	1.35	1.35	1.00	1.00	35
	AR974-2	0.73	0.73	1.39	1.40	1.00	1.00	20
DR5	PP	1.00	4.49	1	3.56	3.56	0.71	n.d.
	AR974-0.25	1.34	6.02	1.43	5.10	4.22	0.84	173
	AR974-0.5	0.98	4.40	1.53	5.46	3.85	0.77	106
	AR974-1	1.17	5.25	1.41	5.02	3.71	0.74	41
	AR974-2	0.90	4.05	1.06	3.77	2.70	0.54	3
DR10	PP	1.00	11.34	1	11.04	11.04	1.10	n.d.
	AR974-0.25	1.22	13.80	1.42	15.63	12.93	1.29	166
	AR974-0.5	1.11	12.59	1.63	17.96	12.68	1.27	125
	AR974-1	0.96	10.86	1.07	11.88	8.77	0.88	8
	AR974-2	0.82	9.33	0.77	8.54	6.12	0.61	−11
DR15	PP	1.00	14.89	1	16.42	16.42	1.09	n.d.
	AR974-0.25	0.99	14.73	1.05	17.29	14.31	0.95	21
	AR974-0.5	0.99	14.77	1.20	19.60	13.84	0.92	39
	AR974-1	1.00	14.90	1.03	16.88	12.46	0.83	3
	AR974-2	0.90	13.43	0.82	13.54	9.70	0.65	−9

n.d.: not defined (*f* = 0).

**Table 5:** Analysis of variance of tensile modulus (Series II) versus draw ratio and fumed silica (AR974). See other data in Supplementary Material.

Source	DF	Adj SS	Adj MS	*F*-Value	*p*-Value
Model	3	179.740	59.913	56.00	0.000
Linear	2	147.350	73.675	68.87	0.000
Draw Ratio	1	143.097	143.097	133.76	0.000
AR974	1	3.508	3.508	3.28	0.089
2-Way Interactions	3	2.131	2.131	1.99	0.177
Draw Ratio x AR974	1	2.131	2.131	1.99	0.177
Error	16	17.117	1.070		
Total	19	196.857			

**Table 6:** Elastic (*E*_M_ and *E*_K_) and viscous (η_M_ and η_K_) parameter of the Burgers model characterizing the creep compliance of polypropylene/fumed silica nanocomposite fibers in isothermal creep tests at 30 °C (σ_0_ = 3 MPa).

Fiber	*E*_M_ (Gpa)	η_M_ (GPa·s)	*E*_K_ (Gpa)	η_K_ (GPa·s)	*R*^2^
DR = 1					
PP	0.925 ± 0.003	284 ± 25	1.45 ± 0.04	4524 ± 230	0.987
AR974-0.25	0.590 ± 0.005	197 ± 25	1.09 ± 0.05	3831 ± 240	0.982
AR974-0.5	0.793 ± 0.003	221 ± 22	1.35 ± 0.04	4840 ± 186	0.983
AR974-1	0.993 ± 0.002	294 ± 17	1.69 ± 0.04	5917 ± 139	0.981
AR974-2	0.981 ± 0.003	358 ± 22	1.56 ± 0.03	5701 ± 160	0.981
DR = 10					
PP	2.4 ± 0.4	684 ± 10	9.1 ± 0.1	44,326 ± 236	0.972
AR974-0.25	4.8 ± 0.2	697 ± 3	16.7 ± 0.2	310,560 ± 790	0.968
AR974-0.5	6.3 ± 0.1	501 ± 6	20.3 ± 0.2	128,040 ± 932	0.978
AR974-1	2.8 ± 0.3	1152 ± 7	11.3 ± 0.5	52,994 ± 189	0.970
AR974-2	3.3 ± 0.4	846 ± 10	10.2 ± 0.4	59,572 ± 189	0.981
DR = 15					
PP	2.2 ± 0.4	548 ± 78	8.3 ± 0.5	89,525 ± 224	0.967
AR974-0.25	6.2 ± 0.8	704 ± 27	23.8 ± 0.2	228,832 ± 404	0.964
AR974-0.5	7.0 ± 0.8	1051 ± 41	27.0 ± 0.2	1,075,268 ± 561	0.962
AR974-1	3.1 ± 0.5	689 ± 86	8.6 ± 0.4	95,419 ± 205	0.973
AR974-2	5.5 ± 0.6	962 ± 54	22.7 ± 0.2	115,080 ± 808	0.967

**Table 7:** Selected TGA results of neat PP and nanofilled PP fibers.

Fiber	Temperature	of selected	Mass loss	DTGA	Maximum	Residual
	−10% *T*_0.1_ (°C)	−50% *T*_0.5_ (°C)	−80% *T*_0.8_ (°C)	peak (°C)	degradation rate (−%/°C)	mass at 600°C (%)
PP	267 ± 2	301 ± 3	328 ± 2	300	−1.54	0.0 ± 0.0
AR974-0.25	274 ± 2	310 ± 3	329 ± 2	316	−1.43	0.5 ± 0.1
AR974-0.5	276 ± 3	312 ± 3	333 ± 2	319	−1.73	0.8 ± 0.1
AR974-1	280 ± 2	317 ± 3	338 ± 2	330	−1.44	1.2 ± 0.3
AR974-2	274 ± 2	324 ± 2	355 ± 3	328	−1.09	3.3 ± 0.2

**Table 8:** Crystallinity content and intensity ratio of the PP nanocomposite calculated from XRD measurements.

DR	Fiber	*X*c (%)	*I*(040)/*I*(040)_ref_	*I*(130)/*I*(130)_ref_
DR = 1	PP	23.6	1.0	1.0
	AR974-0.25	24.1	1.0	1.0
	AR974-0.5	23.5	1.0	1.1
	AR974-1	27.1	1.2	1.1
	AR974-2	27.6	1.7	1.1
DR = 10	PP	57.7	3.1	4.1
	AR974-0.25	57.8	3.4	4.0
	AR974-0.5	48.1	3.1	3.9
	AR974-1	53.7	3.4	4.4
	AR974-2	56.4	3.1	4.6
DR = 15	PP	55.7	2.1	2.6
	AR974-0.25	53.9	2.8	3.4
	AR974-0.5	52.4	3.1	3.3
	AR974-1	48.5	3.1	4.1
	AR974-2	50.0	2.5	3.1

**Table 9:** Crystallite size dimensions (nm) of PP nanocomposite evaluated from XRD spectra.

Fiber	Reflex (hkl)	DR = 1	DR = 10	DR = 15
PP	(110)	3 (overlapped)	8.9 ± 0.2	9.1 ± 0.2
	(040)		8.7 ± 0.4	7.7 ± 0.4
	(130)		8.0 ± 0.3	7.8 ± 0.7
AR974-0.25	(110)	4.9 ± 0.5	9.5 ± 0.2	8.7 ± 0.2
	(040)	3.0 ± 0.7	8.8 ± 0.4	8.6 ± 0.4
	(130)	5.5 ± 0.6	7.3 ± 0.3	6.8 ± 0.3
AR974-0.5	(110)	4.8 ± 0.5	8.7 ± 0.2	9.8 ± 0.2
	(040)	3 ± 1	8.5 ± 0.4	9.2 ± 0.5
	(130)	5.2 ± 0.6	7.6 ± 0.4	8.1 ± 0.3
AR974-1	(110)	6.5 ± 0.7	8.9 ± 0.2	8.2 ± 0.2
	(040)	3 ± 1	8.0 ± 0.4	6.3 ± 0.4
	(130)	5.2 ± 0.4	8.1 ± 0.6	7.6 ± 0.7
AR974-2	(110)	9.8 ± 0.8	10.2 ± 0.2	8.5 ± 0.2
	(040)	12.7 ± 0.6	9.8 ± 0.4	8.0 ± 0.7
	(130)	10 ± 1	9.7 ± 0.3	8.1 ± 0.6

**Table 10:** Tensile storage modulus of neat and nanofilled PP fibers at selected temperatures for various draw ratios (DR).

DR	Fiber	Storage modulus at selected temperatures *T* (GPa)				
		*T* = −100 °C	*T* = −50 °C	*T* = 0 °C	*T* = 50 °C	*T* = 100 °C
DR = 1	PP	3.86	3.22	2.10	0.50	0.15
	AR974-0.25	3.91	3.29	2.12	0.45	0.14
	AR974-0.5	4.01	3.36	2.15	0.52	0.13
	AR974-1	4.31	3.66	2.42	0.71	0.17
	AR974-2	5.04	4.21	2.49	0.80	0.28
DR = 10	PP	22.02	16.72	10.85	6.24	2.06
	AR974-0.25	28.94	23.51	17.51	11.16	4.72
	AR974-0.5	29.47	22.98	15.91	9.43	3.10
	AR974-1	24.01	19.38	12.32	7.30	2.72
	AR974-2	19.88	15.00	8.86	4.64	1.66
DR = 15	PP	27.74	22.70	17.33	11.68	5.34
	AR974-0.25	29.18	24.00	18.50	12.55	6.35
	AR974-0.5	32.51	27.04	20.80	14.32	6.78
	AR974-1	32.65	27.18	20.37	13.85	6.20
	AR974-2	28.17	23.28	16.90	12.12	5.91

**Table 11:** Temperature and height of the loss modulus (*T*_β_) and (*T*_α_) peaks of neat and nanofilled PP fibers as a function of the fumed silica volume fraction. Effective volume fraction of dispersed phase, (ф_e_) and B parameter were calculated according to the model proposed by Sumita et al. [63].

Material	*T*_β_/(°C)	*E″* Peak (MPa)	*T*_α_/*E*″ Peak (°C)/(MPa)		ф_e_		*B*	
	DR = 10	DR = 15	DR = 10	DR = 15	DR = 10	DR = 15	DR = 10	DR = 15
PP	−28/1646	−25/1408	67/706	69/1378	-	-	-	-
AR974-0.25	−27/1556	−24/1568	65/1368	71/1492	0.482	0.087	193.0	34.9
AR974-0.5	−27/1874	−22/1603	64/1164	69/1650	0.392	0.175	78.4	35.1
AR974-1	−20/1618	−25/2115	73/748	70/1841	0.053	0.253	5.3	25.3
AR974-2	−19/1528	−22/1689	74/410	76/1331	−0.70	−0.013	−35.1	−0.6

## Supplementary Materials

The following are available online at www.mdpi.com/2073-4360/9/2/41/s1. Table S1: Experimental array of Series I. Tensile modulus and stress at break of PP fibers produced at different draw ratios, fumed silica contents (AR974) and compatibilizer contents (C). Table S2: Series I. Analysis of variance of tensile modulus (Series I) versus draw ratio, fumed silica (AR974) and compatibilizer (C). Table S3: Analysis of tensile modulus (Series I). Coded coefficients, Regression Equation and Alias Structure. Table S4: Analysis of tensile modulus (Series I). Fits and diagnostics for unusual observations. Figure S1: Series I. Contour plot of tensile modulus [GPa] vsersus draw ratio and AR974 at constant C 0.5% by vol. Figure S2: Series I. Contour plot of tensile modulus [GPa] versus draw ratio and C at constant AR974 0.25% by vol. Table S5: Series I. Analysis of variance of stress at break versus draw ratio, fumed silica (AR974) and compatibilizer (C). Table S6: Series I. Analysis of stress at break. Coded coefficients, regression equation and alias structure. Table S7: Series I. Analysis of stress at break. Fits and diagnostics for unusual observations. Figure S3: Series I. Contour plot of stress at break [MPa] versus draw ratio and AR974 at constant C 0.5% by vol. Figure S4: Series I. Contour plot of stress at break [MPa] versus draw ratio and C at constant AR974 0.25% by vol. Table S8: Experimental array of Series II. Tensile modulus, tenacity, and stress at break of PP fibers produced at different draw ratios and fumed silica contents (AR974). Table S9: Series II. Analysis of variance of tensile modulus versus draw ratio and fumed silica (AR974). Table S10: Series II. Analysis of tensile modulus. Coded coefficients, regression equation and alias structure. Table S11: Series II. Analysis of tensile modulus. Fits and diagnostics for nuusual observations. Figure S5: Series II. Normal plot of the standardized effects for tensile modulus [GPa]. Figure S6: Interaction plot for tensile modulus [GPa] in Series II. Figure S7: Main effects plot for tensile modulus [GPa] in Series II. Table S12: Series II. Analysis of variance of tenacity versus draw ratio and fumed silica (AR974). Table S13: Series II. Analysis of tenacity. Coded coefficients, regression equation and alias structure. Table S14: Series II. Analysis of tenacity. Fits and diagnostics for unusual observations. Figure S8: Series II. Normal plot for tenacity [cN/tex]. Figure S9: Interaction plot for tenacity [cN/tex] in Series II. Figure S10: Main effects plot for tenacity [cN/tex] in Series II. Table S15: Series II. Analysis of variance of stress at break versus draw ratio and fumed silica (AR974). Table S16: Series II. Analysis of stress at break. Coded coefficients, regression equation and alias structure. Table S17: Series II. Analysis of stress at break Fits and diagnostics for unusual observations. Figure S11: Series II. Normal plot for stress at break [MPa]. Figure S12: Interaction plot for stress at break [MPa] in Series II. Figure S13: Main effects plot for stress at break [MPa] in Series II.
