# Supplementary material for: Effects of Fumed Silica and Draw Ratio on Nanocomposite Polypropylene Fibers
Source: Polymers (Basel). 2017 Jan 28;9(2):41. doi: 10.3390/polym9020041 (PMC6432459; doi:10.3390/polym9020041)
Supplement: Supplementary file 1 [file polymers-09-00041-s001.pdf]

Supplementary Material: Effects of Fumed Silica and Draw Ratio on Nanocomposite Polypropylene Fibers

Luca Fambri, Izabela Dabrowska, Riccardo Ceccato and Alessandro Pegoretti

1. Statistical Analysis of Series I (Minitab 17 Software, Release 3.1.0)

Table S1. Experimental array of Series I. Tensile modulus and stress at break of PP fibers produced at different draw ratios, fumed silica contents (AR974) and compatibilizer contents (C).

Run	Experimental	Factors		Tensile Modulus	Stress at Break
No.	Draw Ratio	AR974	C	[GPa]	[MPa]
1	1	0	0	0.48	83
2	4	0	0	1.55	289
3	5	0	0	1.7	374
4	7	0	0	2.5	522
5	10	0	0	5.3	944
6	12	0	0	6.5	1077
7	15	0	0	7.88	1240
8	17.4	0	0	8.5	1230
9	1	0	0.5	0.62	74.4
10	5	0	0.5	1.9	332
11	8.6	0	0.5	4.6	872
12	10	0	0.5	5.6	932
13	13.7	0	0.5	8.12	1058
14	15	0	0.5	8.8	1086
15	16	0	0.5	9.5	1125
16	20.5	0	0.5	11.5	1203
17	1	0	1	0.61	70
18	3.7	0	1	1.55	250
19	5	0	1	2	320
20	9.2	0	1	5.24	876
21	10	0	1	5.9	1068
22	12.8	0	1	8.5	1187
23	15	0	1	9.7	1213
24	17.4	0	1	10.5	1256
25	1	0.5	0	0.68	70
26	4	0.5	0	2.18	250
27	5	0.5	0	2.62	369
28	7	0.5	0	3.5	576
29	8	0.5	0	5.6	890
30	9	0.5	0	8.22	940
31	10	0.5	0	8.62	1054
32	12.8	0.5	0	9.15	1185
33	15	0.5	0	9.41	1237
34	1	0.5	0.5	0.6	70
35	4	0.5	0.5	1.55	267
36	5	0.5	0.5	1.79	370
37	6.25	0.5	0.5	2.1	497
38	7.3	0.5	0.5	2.6	644
39	8.2	0.5	0.5	4	772
40	10	0.5	0.5	5.8	976
41	13.7	0.5	0.5	8.4	1183
42	15	0.5	0.5	9.26	1211
43	1	0.5	1	0.66	70
44	4.3	0.5	1	1.78	398
45	5	0.5	1	2.44	485
46	8.6	0.5	1	5.2	933
47	10	0.5	1	7.04	1015
48	12	0.5	1	8.95	1118

1.1. Factorial Regression: Tensile Modulus [GPa] versus Draw Ratio; AR974; C

Table S2. Series I. Analysis of variance of tensile modulus versus draw ratio, fumed silica (AR974) and compatibilizer (C).

Source	DF	Adj SS	Adj MS	*F*-Value	*p*-Value
Model	7	501.127	71.590	95.29	0.000
Linear	3	465.500	155.167	206.54	0.000
Draw Ratio	1	439.247	439.247	584.67	0.000
AR974	1	7.206	7.206	9.59	0.004
C	1	0.451	0.451	0.60	0.443
2-Way Interactions	3	9.689	3.230	34.30	0.010
Draw Ratio x AR974	1	2.835	2.835	3.77	0.059
Draw Ratio x C	1	0.436	0.436	0.58	0.451
AR974 x C	1	4.336	4.336	5.77	0.021
3-Way Interactions	1	0.703	0.703	0.94	0.339
Draw Ratio x AR974 x C	1	0.703	0.703	0.94	0.339
Error	40	30.051	0.751		
Total	47	531.178			

Model summary: $S = 0.866763$; R-sq = 94.34%; R-sq(adj) = 93.35%; R-sq(pred) = 91.75%.

Table S3. Series I. Analysis of tensile modulus. Coded coefficients, regression equation and alias structure.

Term	Effect	Coef	SE Coef	*T*-Value	*p*-Value	VIF
Constant		6.397	0.147	43.61	0.000	
Draw Ratio	12.831	6.415	0.265	24.18	0.000	1.20
AR974	0.909	3.10	0.454	0.147	0.004	1.37
C	0.292	0.146	0.188	0.77	0.443	1.45
Draw Ratio x AR974	1.031	0.515	0.265	1.94	0.059	1.34
Draw Ratio x C	0.524	0.262	0.344	0.76	0.451	1.48
AR974 x C	−0.905	−0.452	0.344	−2.40	0.021	1.45
Draw Ratio x AR974 x C	−0.665	−0.332	0.188	−0.97	0.339	1.48
**Regression Equation**	**in Uncoded Units**					
Tensile Modulus [GPa] =	−0.506 + (0.5442 Draw Ratio − 0.11 AR974 − 0.114 C + 0.348 Draw Ratio x AR974 + 0.1219 Draw Ratio x C − 0.69 AR974 x C − 0.273 Draw Ratio x AR974 x C					
**Alias Structure**	**Factor Name ***					
Linear	A, B, C					
2-Way Interactions	AB, AC, BC					

**A* = Draw Ratio; B = AR974; C = Compatibilizer

Table S4. Series I. Analysis of tensile modulus. Fits and diagnostics for unusual observations.

Obs	Tensile Modulus [GPa]	Fit	Resid	Std Resid
30	8.220	5.901	2.319	2.82 R
31	8.600	6.619	1.981	2.43 R
38	2.600	4.399	−1.799	−2.12 R
48	8.920	7.424	1.496	2.12 R

R: Large residual.

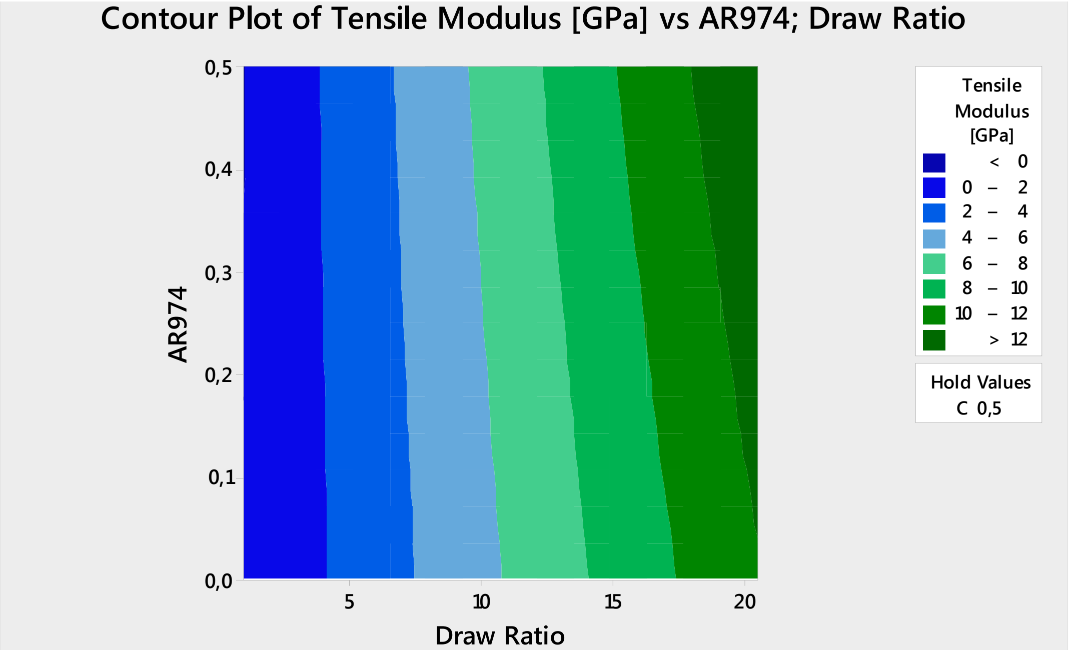

Figure S1. Series I. Contour plot of tensile modulus [GPa] versus draw ratio and AR974 at constant C 0.5% by vol.

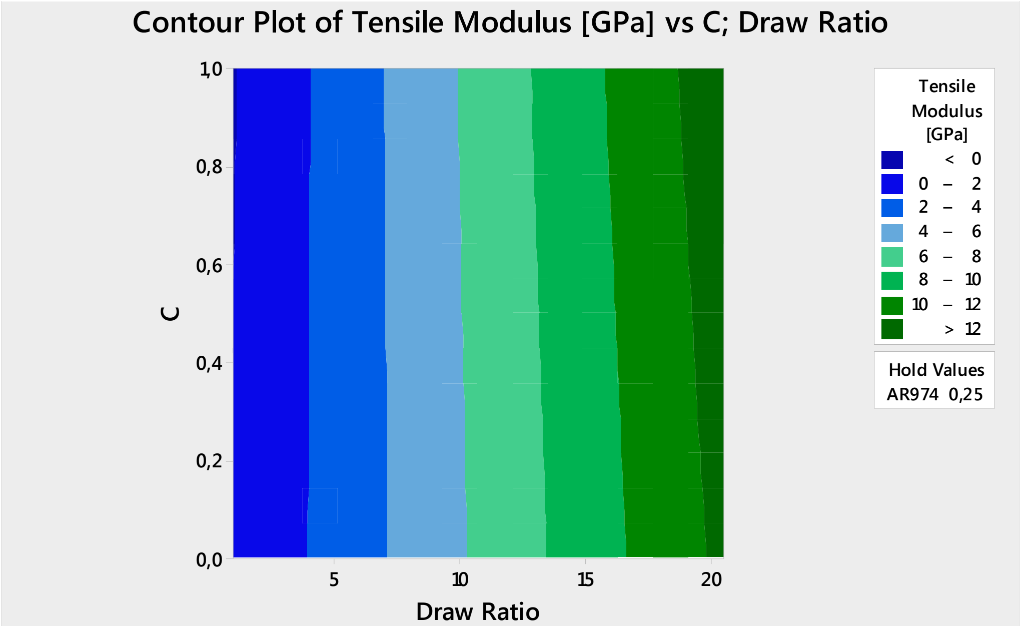

Figure S2. Series I. Contour plot of tensile modulus [GPa] versus draw ratio and C at constant AR974 0.25% by vol.

1.2. Factorial Regression: Stress at Break [MPa] versus Draw Ratio; AR974; C

Table S5. Series I. Analysis of variance of stress at break versus draw ratio, fumed silica (AR974) and compatibilizer (C).

Source	DF	Adj SS	Adj MS	*F*-Value	*p*-Value
Model	7	7,469,665	1,067,095	68.49	0.000
Linear	3	7,194,005	2,398,002	153.91	0.000
Draw Ratio	1	6,870,401	6,870,401	440.95	0.000
AR974	1	165,177	165,177	10.60	0.002
C	1	9030	9030	0.58	0.451
2-Way Interactions	3	117,151	39,050	2.51	0.073
Draw Ratio * AR974	1	114,857	114,857	7.37	0.010
Draw Ratio * C	1	830	830	0.05	0.819
AR974 * C	1	2	2	0.00	0.990
3-Way Interactions	1	229	229	0.01	0.904
Draw Ratio * AR974 * C	1	229	229	0.01	0.904
Error	40	623,229	15,581		
Total	47	8,092,894			

Model summary: $S = 124.823$; R-sq = 92.30%; R-sq(adj) = 90.95%; R-sq(pred) = 89.13%.

Table S6. Series I. Analysis of stress at break. Coded coefficients, regression equation and alias structure.

Term	Effect	Coef	SE Coef	*T*-Value	*p*-Value	VIF
Constant		914.7	21.1	43.30	0.000	
Draw Ratio	1604.7	802.3	38.2	21.00	0.000	1.20
AR974	137.6	68.8	21.1	3.26	0.002	1.37
C	41.3	20.6	27.1	0.76	0.451	1.45
Draw Ratio x AR974	207.5	103.7	38.2	2.72	0.010	1.34
Draw Ratio x C	22.9	11.4	49.5	0.23	0.819	1.48
AR974 x C	−0.7	−0.3	27.1	−0.01	0.990	1.45
Draw Ratio x AR974 x C	−12.0	−6.0	49.5	−0.12	0.904	1.48

Regression Equation	in Uncoded Units
Stress at break [MPa] =	73.9 + 69.86 Draw Ratio − 208 AR974 + 4 C + 45.0 Draw Ratio x AR974 + 3.6 Draw Ratio x C + 50 AR974 x C − 4.9 Draw Ratio x AR974 x C

Alias Structure	Factor Name *
Linear	A, B, C
2-Way Interactions	AB, AC, BC
3-Way Interactions	ABC

* A = Draw Ratio; B = AR974; C = Compatibilizer

Table S7. Series I. Analysis of stress at break. Fits and diagnostics for unusual observations.

Obs	Stress at break [MPa]	Fit	Resid	Std resid
16	1203.0	1544.5	−341.5	−3.04 R
21	1068.0	811.8	256.2	2.17 R

R: Large residual.

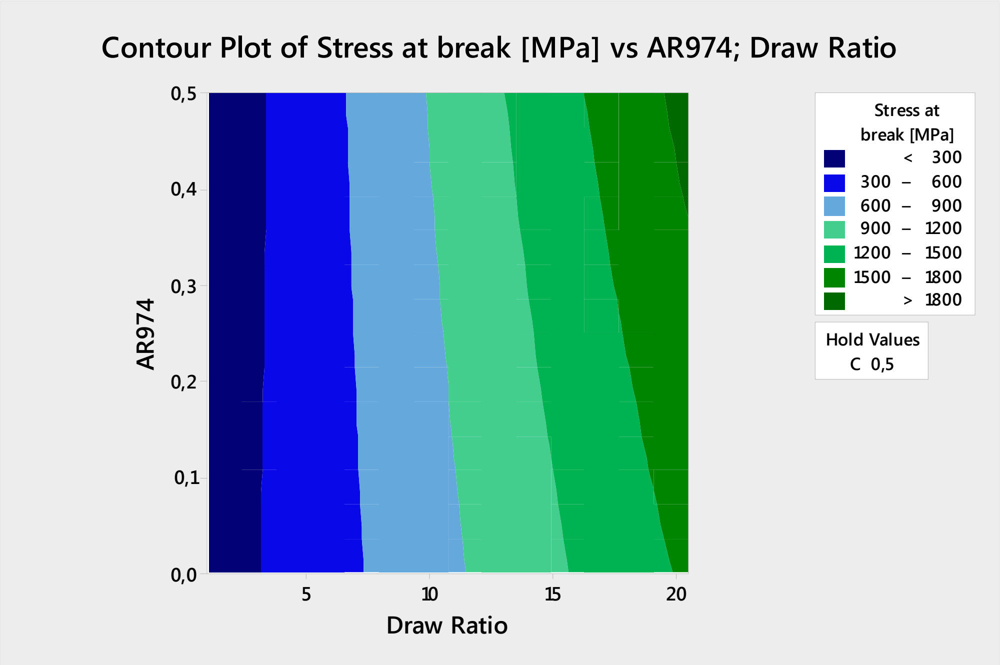

Figure S3. Series I. Contour plot of stress at break [MPa] versus draw ratio and AR974 at constant C 0.5% by vol.

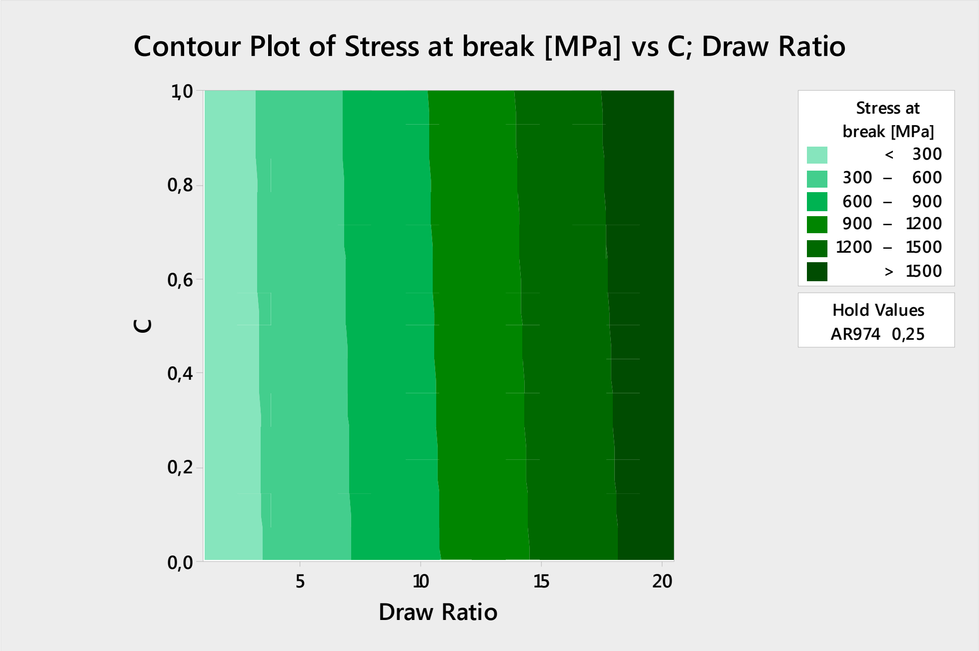

Figure S4. Series I. Contour plot of stress at break [MPa] versus draw ratio and C at constant AR974 0.25% by vol.

2. Statistical Analysis of Series II (Minitab 17 Software, Release 3.1.0)

Table S8. Experimental array of Series II. Tensile modulus, tenacity, and stress at break of PP fibers produced at different draw ratios and fumed silica contents (AR974).

Run	Experimental	Factors	Tensile Modulus	Tenacity	Stress at Break
No.	Draw Ratio	AR974	[GPa]	[cN/tex]	[MPa]
1	1	0	0.48	9.2	83
2	1	0.25	0.58	8.2	74
3	1	0.5	0.68	7.7	70
4	1	1	0.65	9.1	83
5	1	2	0.67	6.7	62
6	5	0	1.71	41.3	374
7	5	0.25	2.45	55.4	503
8	5	0.5	2.62	40.5	369
9	5	1	2.41	48.3	442
10	5	2	1.81	37.3	346
11	10	0	5.3	104.3	944
12	10	0.25	7.5	127	1153
13	10	0.5	8.62	115.8	1054
14	10	1	5.7	99.9	915
15	10	2	4.1	85.8	795
16	15	0	7.88	137	1240
17	15	2.5	8.3	135.5	1230
18	15	0.5	9.41	135.9	1237
19	15	1	8.1	137.1	1256
20	15	2	6.5	123.6	1145

2.1. Factorial Regression: Tensile Modulus [GPa] versus *Draw Ratio; AR974; C*

Table S9. Series II. Analysis of variance of tensile modulus versus draw ratio and fumed silica (AR974).

Source	DF	Adj SS	Adj MS	*F*-Value	*p*-Value
Model	3	179.740	59.913	56.00	0.000
Linear	2	147.350	73.675	68.87	0.000
Draw Ratio	1	143.097	143.097	133.76	0.000
AR974	1	3.508	3.508	3.28	0.089
2-Way Interactions	3	2.131	2.131	1.99	0.177
Draw Ratio x AR974	1	2.131	2.131	1.99	0.177
Error	16	17.117	1.070		
Total	19	196.857			

Model summary: S = 1.03430; R-sq = 91.31%; R-sq(adj) = 89.67%; R-sq(pred) = 88.15%.

Table S10. Series II. Analysis of tensile modulus. Coded coefficients, regression equation and alias structure.

Term	Effect	Coef	SE Coef	*T*-Value	*p*-Value	VIF
Constant		4.266	0.246	17.37	0.000	
Draw Ratio	7.549	3.774	0.326	11.57	0.000	1.13
AR974	−1.186	−0.593	0.327	−1.81	0.089	1.00
Draw Ratio x AR974	−1.228	−0.614	0.435	−1.41	0.177	1.13
**Regression Equation**			**in Uncoded Units**			

Tensile Modulus [GPa] =	−0.157 +0.6269 Draw Ratio +0.109 AR974 − 0.0877 Draw Ratio x AR974
**Alias Structure**	**Factor Name ***
Linear	A, B
2-Way Interactions	AB

* A = Draw Ratio; B = AR974.

Table S11. Series II. Analysis of tensile modulus. Fits and diagnostics for unusual observations.

Obs	Tensile Modulus [GPa]	Fit	Resid	Std Resid
13	8.620	5.728	2.892	2.89 R

R: Large residual.

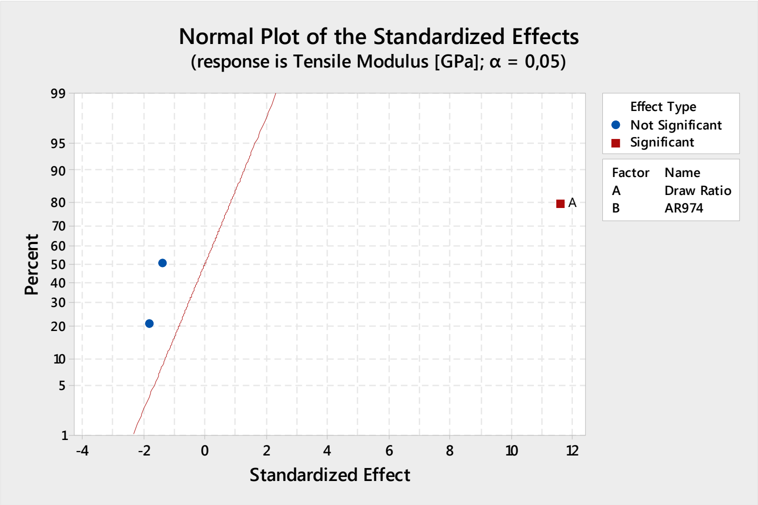

Figure S5. Series II. Normal plot of the standardized effects for tensile modulus [GPa].

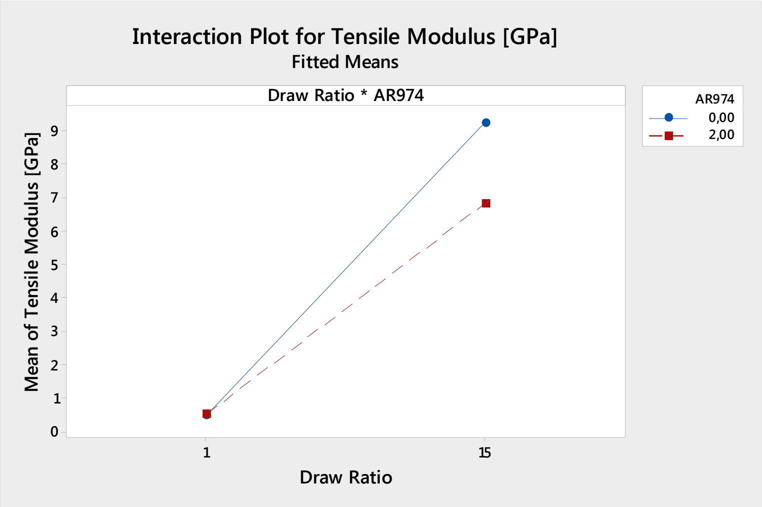

Figure S6. Interaction plot for tensile modulus [GPa] in Series II.

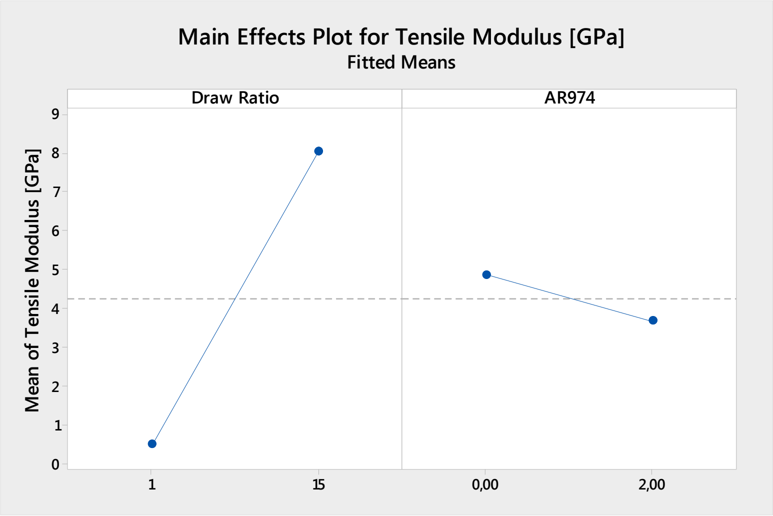

Figure S7. Main effects plot for tensile modulus [GPa] in Series II.

2.2. Factorial Regression: Tenacity [cN/tex] versus Draw Ratio; AR974

Table S12. Series II. Analysis of variance of tenacity versus draw ratio and fumed silica (AR974).

Source	DF	Adj SS	Adj MS	F-Value	*p*-Value
Model	3	48632.8	16210.9	138.23	0.000
Linear	2	42073.4	21036.7	179.38	0.000
Draw Ratio	1	41464.0	41464.0	353.56	0.000
AR974	1	460.6	460.6	3.93	0.065
2-Way Interactions	3	89.3	89.3	0.76	0.396
Draw Ratio x AR974	1	89.3	89.3	0.76	0.396
Error	16	1876.4	117.3		
Total	19	50509.2			

Model Summary: S = 10.8293; R-sq = 96.29%; R-sq(adj) = 95.59%; R-sq(pred) = 94.99%.

Table S13. Series II. Analysis of tenacity. Coded coefficients, regression equation and alias structure.

Term	Effect	Coef	SE Coef	T-Value	*p*-Value	VIF
Constant		73.91	2.57	28.74	0.000	
Draw Ratio	128.49	64.25	3.42	18.80	0.000	1.13
AR974	−13.59	−6.79	3.43	−1.98	0.065	1.00
Draw Ratio x AR974	è7 -7.95	−3.97	4.56	−0.87	0.396	1.13

**Regression Equation**	**in Uncoded Units**
Tenacity[cN/tex] =	2.74 + 9.746 Draw Ratio − 2.25 AR974 − 0.568 Draw Ratio x AR974

**Alias Structure**	**Factor Name ***
Linear	A, B
2-Way Interactions	AB

* A= Draw Ratio; B = AR974.

Table S14. Series II. Analysis of tenacity. Fits and diagnostics for unusual observations.

Obs	Tenacity [cN/tex]	Fit	Resid	Std Resid
12	127.00	98.22	28.78	2.78 R

R: Large residual.

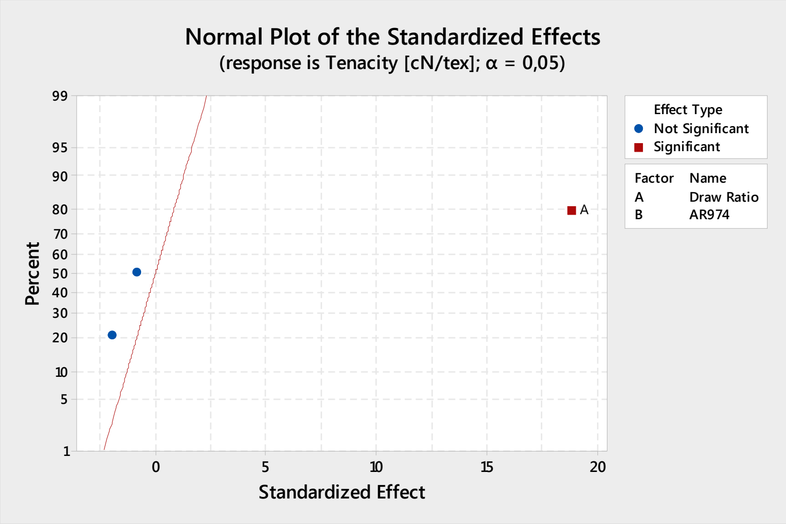

Figure S8. Series II. Normal plot for tenacity [cN/tex].

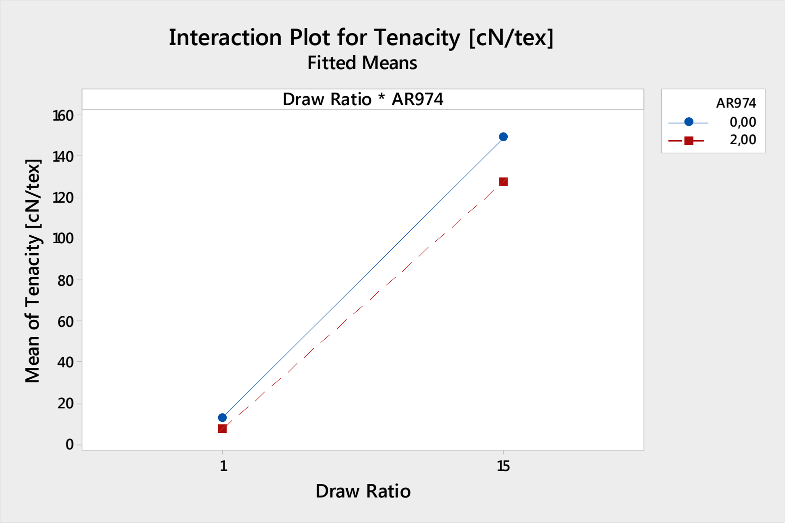

Figure S9. Interaction plot for tenacity [cN/tex] in Series II.

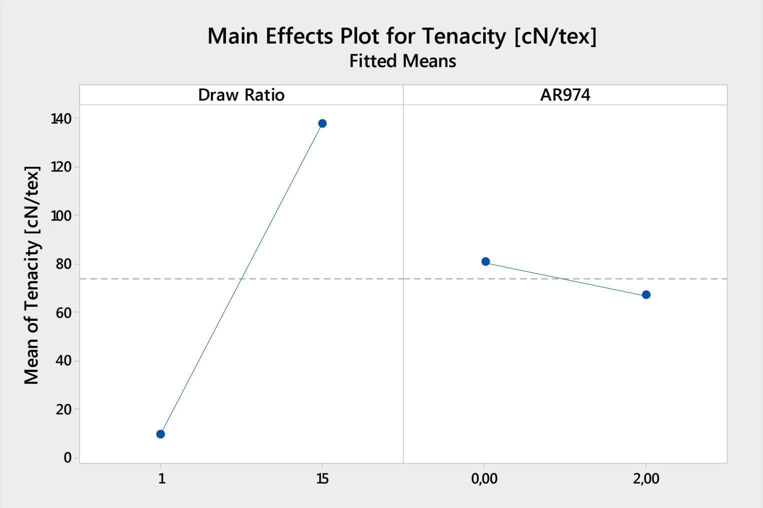

Figure S10. Main effects plot for tenacity [cN/tex] in Series II.

2.3. Factorial Regression: Stress at Break [MPa] versus *Draw Ratio; AR974*

Table S15. Series II. Analysis of variance of stress at break versus draw ratio and fumed silica (AR974).

Source	DF	Adj SS	Adj MS	*F*-Value	*p*-Value
Model	3	4,042,361	1,347,454	138.94	0.000
Linear	2	3,515,800	1,757,900	181.26	0.000
Draw Ratio	1	3,475,150	3,475,150	358.33	0.000
AR974	1	29,620	29,620	3.05	0.100
2-Way Interactions	3	4981	4981	0.51	0.484
Draw Ratio x AR974	1	4981	4981	0.51	0.484
Error	16	155,173	9698		
Total	19	4,197,534			

Model summary: *S* = 98.4800; *R*-sq = 96.30%; *R*-sq(adj) = 95.61%; *R*-sq(pred) = 95.00%.

Table S16. Series II. Analysis of stress at break. Coded coefficients, regression equation and alias structure.

Term	Effect	Coef	SE Coef	*T*-Value	*p*-Value	VIF
Constant		676.4	23.4	28.93	0.000	
Draw Ratio	1176.3	588.2	31.1	18.93	0.000	1.13
AR974	−109.0	−54.5	31.2	−1.75	0.100	1.00
Draw Ratio x AR974	−59.4	−29.7	41.4	−0.72	0.484	1.13
**Regression Equation**			**in Uncoded Units**			
Tenacity [MPa] =			24.8 + 88.27 Draw Ratio − 20.6 AR974 − 4.24 Draw Ratio x AR974			
**Alias Structure**			**Factor Name ***			
Linear			A, B			
2-Way Interactions			AB			

* A = Draw Ratio; B = AR974.

Table S17. Series II. Analysis of stress at break Fits and diagnostics for unusual observations.

Obs	Stress at break [MPa]	Fit	Resid	Std Resid
12	1153.0	891.7	261.3	2.78 R

R: Large residual.

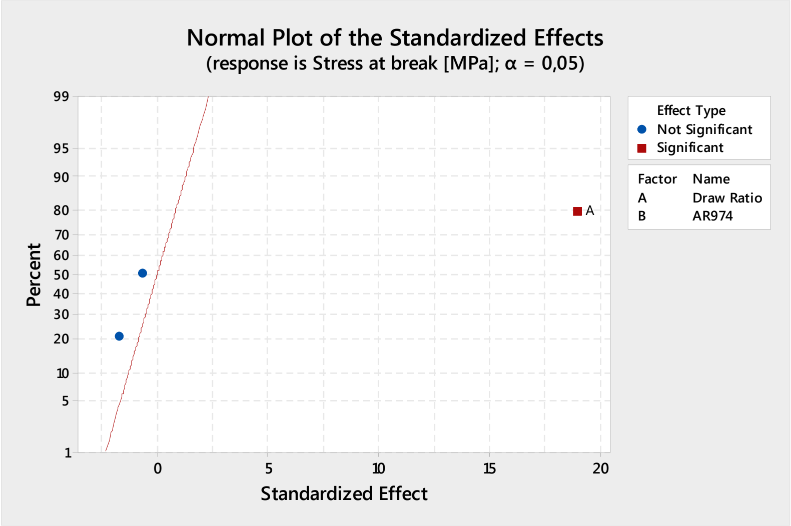

Figure S11. Series II. Normal plot for stress at break [MPa].

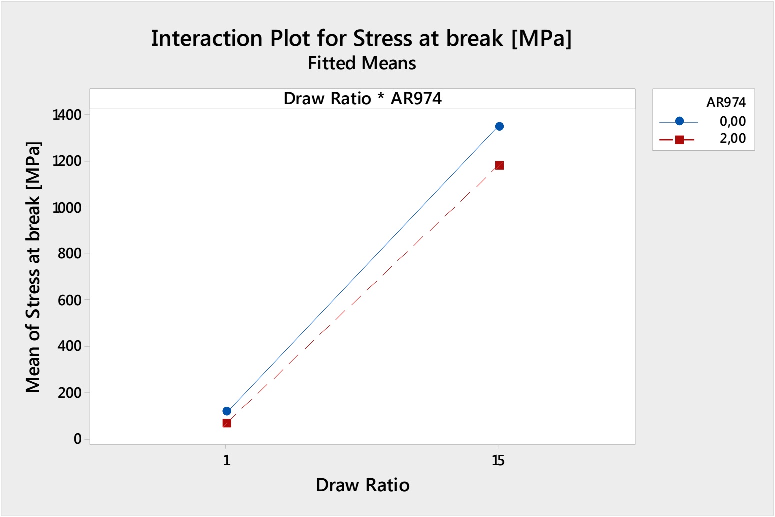

Figure S12. Interaction plot for stress at break [MPa] in Series II.

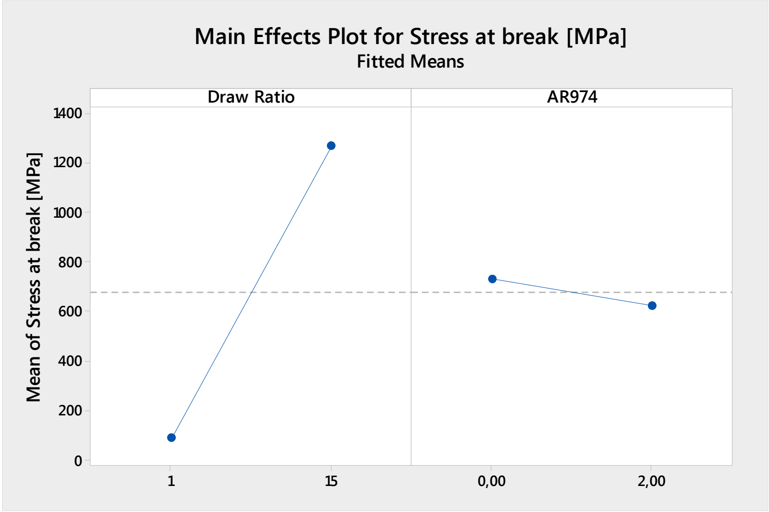

Figure S13. Main effects plot for stress at break [MPa] in Series II.
